# Remembering St. Louis Individual—structural violence and acute bacterial infections in a historical anatomical collection

**DOI:** 10.1038/s42003-022-03890-z

**Published:** 2022-10-03

**Authors:** Rita M. Austin, Molly Zuckerman, Tanvi P. Honap, Hedwig Lee, Geoff K. Ward, Christina Warinner, Krithivasan Sankaranarayanan, Courtney A. Hofman

**Affiliations:** 1 University of Oklahoma, Laboratories of Molecular Anthropology and Microbiome Research, Norman, OK 73019 USA; 2 University of Oklahoma, Department of Anthropology, Norman, OK 73019 USA; 3 Smithsonian Institution, National Museum of Natural History, Department of Anthropology, Washington, DC 20560 USA; 4 Mississippi State University, Department of Anthropology and Middle Eastern Cultures, Mississippi State, MS 39762 USA; 5 Washington University in St. Louis, Department of Sociology, St. Louis, MO 63130 USA; 6 Duke University, Department of Sociology, Durham, NC 27708 USA; 7 Washington University in St. Louis, African and African-American Studies, St. Louis, MO 63130 USA; 8 Harvard University, Department of Anthropology, Cambridge, MA 02138 USA; 9 University of Oklahoma, Department of Microbiology and Plant Biology, Norman, OK 73019 USA; 10 Present Address: Natural History Museum, University of Oslo, Oslo, 0318 Norway

**Keywords:** Bacterial infection, Bacterial genetics, Ethics, Microbiome, History

## Abstract

Incomplete documentary evidence, variable biomolecular preservation, and limited skeletal responses have hindered assessment of acute infections in the past. This study was initially developed to explore the diagnostic potential of dental calculus to identify infectious diseases, however, the breadth and depth of information gained from a particular individual, St. Louis Individual (St.LI), enabled an individualized assessment and demanded broader disciplinary introspection of ethical research conduct. Here, we document the embodiment of structural violence in a 23-year-old Black and/or African American male, who died of lobar pneumonia in 1930s St. Louis, Missouri. St.LI exhibits evidence of systemic poor health, including chronic oral infections and a probable tuberculosis infection. Metagenomic sequencing of dental calculus recovered three pre-antibiotic era pathogen genomes, which likely contributed to the lobar pneumonia cause of death (CoD): *Klebsiella pneumoniae* (13.8X); *Acinetobacter nosocomialis* (28.4X); and *Acinetobacter junii* (30.1X). Ante- and perimortem evidence of St.LI’s lived experiences chronicle the poverty, systemic racism, and race-based structural violence experienced by marginalized communities in St. Louis, which contributed to St.LI’s poor health, CoD, anatomization, and inclusion in the Robert J. Terry Anatomical Collection. These same embodied inequalities continue to manifest as health disparities affecting many contemporary communities in the United States.

## Abstract (web summary)

An investigation into the cause of death of St. Louis Individual, a 23-year old Black or African American male who died in the 1930s, reveals evidence of structural violence and the impact of systemic racism in historically marginalized communities.

## Introduction

### Dedicated to St. Louis Individual, whose partial story we hope to respectfully tell here

Studies of the ecology and evolution of infectious disease, particularly acute infections, have been limited by the biases and incompleteness of related documentary evidence^1,2^, variable biomolecular preservation, and the limited range of possible skeletal tissue responses to physiological insults (i.e., bone proliferation, loss and/or altered morphology), including infection^3^. These factors are exacerbated by variation within pathogen biology, pathogenicity, virulence, and the relatively slow reaction time of bone, whereby many acute infections (e.g., pneumonia, influenza, cholera) do not generate skeletal reactions before death or resolution of infection. In skeletal reference collections (e.g., anatomical collections), which have been fundamental to medical training and to methods development in biological anthropology^4^, these issues are further compounded by sampling bias^5,6^ towards the inclusion of unique, distinctive, or advanced disease presentations^7^. Together, these material restrictions can convolute post-mortem taphonomic changes, affect molecular analyses^8^, impede paleopathological diagnoses, and confuse interpretations of morbidity, frailty, selective mortality, and disease burden in past populations^9^. However, with the emergence of ancient pathogen genomics, infectious diseases are now regularly being identified from ancient contexts and deceased individuals, allowing researchers to explore pathogen evolutionary histories^10^, which can ultimately inform public health responses to current and future disease events^11^.

While individuals exhibiting soft and hard tissue pathologies have regularly been the foci of ancient pathogen research, a viable alternative to more destructive sample types (e.g., petrous portion, dentin) is dental calculus (calcified dental plaque), as it yields human, microbial, pathogen, and dietary biomolecules with limited environmental contamination^12^. Because dental plaque calcifies periodically throughout life, dental calculus has been considered to be a cumulative reservoir of an individual’s oral health and ingested and/or inhaled pathogens^13,14^.

Pneumonia, like influenza, represented a major source of mortality in past populations, especially prior to the mid-20th century in industrializing nations^2^. However, it does not generate skeletal lesions^3^ and is often misclassified in historical records^2^, confounding interpretations of past disease burden. Hospital- and community-acquired bacterial pneumonia can be caused by various bacterial species. Because pneumonatic agents can be transmitted through inhalation or aspiration, regular contact between the respiratory tract and the oral mucosa increases the likelihood of pathogen incorporation into dental calculus, thus potentially permitting the recovery of pathogen DNA.

Integrating archival documentation, skeletal pathology, and shotgun metagenomic analyses of dental calculus, we present an osteobiography^15,16^ revealing ante- and perimortem experiences of an individual from the Smithsonian Institution’s Robert J. Terry Anatomical Collection (Terry). The cause of death (CoD) for Terry Collection individuals is documented in death certificates, allowing for molecular evidence of CoD to be cross-referenced. According to their death certificate, St. Louis Individual (St.LI) was a 23-year-old, Black and/or African American male laborer, who died of lobar pneumonia in St. Louis, Missouri, in the 1930s. To the best of our knowledge, this is the first study to present pneumonia as an investigable disease in historical and bioarchaeological contexts. The assumption that dental calculus is only a long-term reservoir is also questioned^17^, as our study recovered genomic data for pathogens associated with acute, perimortem diseases. Through combined approaches and efforts we are able to provide direct, context-specific insights into the intersection of biological processes (e.g., infectious disease) and social conditions (e.g., race-based structural violence) that shaped the biosocial lived experiences, and health and disease outcomes of a Black and/or African American in St. Louis during the early 20th century. This nuanced approach, which aims to unpack and problematize what is known, is one way of demonstrating respect for individuals whose remains we have the privilege of studying and reaffirms biological anthropology’s commitment to social justice^16^.

### Black and/or African American health and well-being in early 20th century St. Louis

Early 20th century St. Louis was keenly inhospitable to Black and/or African American communities, creating deeply harmful social, economic, biological, and environmental conditions and exposures that produced poor individual health outcomes and population-level health disparities. In conjunction with large-scale, national-level processes of Jim Crow and processes of race-based structural violence, this manifested through social closure, whereby the established, dominant population (i.e., White and/or European American St. Louisans) coordinated social, economic, and political exclusion through monopoly tactics, exploitation, opportunity hoarding, and other means^18^. Social closure, alongside St. Louis’ rapidly increasing population during the Great Migration (c. 1910–1970) from the Deep South, generated increased competition for societal and environmental resources (e.g., food insecurity, increased risk of infectious disease, limited job opportunities, etc.) in already under-resourced and segregated areas, while simultaneously inhibiting any political power to alter these conditions^18,19^. For example, many Black and/or African Americans migrants to St. Louis could have found work opportunities on the riverfront (a major employment sector) before 1900, yet this market rapidly collapsed amid the growth of the railroad industry in the 1930s, during St.LI’s lifetime^20^. Ultimately, these challenging and intersecting conditions may have meant that little to no real economic gain was available for migrants^21^.

St. Louis’ socio-political policies and practices related to health, education, law, housing, etc. exacerbated the hostile social environment faced by Black and/or African Americans. Beginning in the 19th century, St. Louis’ formal and informal residential segregation practices, including the US’s first residential segregation ordinance in 1916, intensively concentrated Black and/or African American residents into largely industrial areas (e.g., the riverfront, central corridor of Mill Creek Valley), where predominantly poor and recent migrants crowded into poor quality housing that often lacked running water and indoor plumbing, creating communities that far exceeded 75% Black and/or African American by population^19,22,23^. These areas were also marked by high levels of air pollution caused by the coal burning industries and domestic heating in the densely populated downtown areas^24^.

In many instances, migrants were internally displaced by racial terror and acts or threats of mass violence (e.g., banishments, threatened lynching) and economic or political persecution (e.g., 1917 East St. Louis race massacre). Indeed, historians^25^ have characterized the period immediately preceding St.LI’s birth (1877–1901) in St. Louis as an unprecedented era of anti-Black political violence and White power politics. Vibrant communities and neighborhoods^23^ were often uprooted, largely to create economic and residential opportunities for White and/or European American communities. These displacements further compounded the economic, biological, and environmental stressors already facing Black and/or African American communities, through ‘root shock’, a traumatic stress reaction associated with repeated displacements that is related to the destruction of the emotional ecosystem. Critically, root shock could impact entire communities, as well as causing intergenerational trauma^26^.

The nation-wide, 19th and early 20th century process of “medical apartheid”^27^ was also present in St. Louis healthcare systems, which were notorious for highly limited and poor-quality treatment for Black and/or African Americans^28^. Black and/or African Americans would often delay, or even forego, medical care due to distrust of healthcare institutions and fear for their safety and well-being, as patient mistreatment, experimentation, and/or the disappearance of their bodies after death was common (see Washington^27^). Intergenerational stressors, coupled with deficient physical and mental health care, further increased risks of mortality^29^ and contributed to detrimental coping behaviors (e.g., smoking, alcohol consumption)^30^. Healthcare systems available to Black and/or African Americans in St. Louis changed over time but were still deeply unequal to those available to White and/or European American residents. Prior to the establishment of St. Louis’ first public hospital for Black and/or African American patients and physicians in 1919, City Hospital #2 (later rebuilt as the Homer G. Phillips Hospital), patients were housed in dingy, unsanitary basement quarters in White and/or European American hospitals, with Black and/or African American physicians forced into assistant roles; the Missouri State Medical Association restricted membership to White and/or European American physicians until the 1940s^31^. Even after 1919, separate and unequal conditions persisted in segregated hospitals, with one observer claiming “sanitary conditions for the animals at Forest Park Zoo are better than those at the Negro hospital”^32^ (see also O’Connor^31^). City Hospital #2 was notoriously under-staffed, cramped, and underfunded, with consistently poor patient outcomes^33–39^. According to his death certificate, St.LI was admitted to this hospital, which may have contributed to his perimortem experiences of infection and premature death and postmortem experiences, including inclusion in the Terry Collection.

### The Terry Collection

Assembled between 1910 and 1967, the Terry Collection is one of the most complete and well-studied documented collections in the world^40–42^. Assembled with the intent of representing human variation, individuals in the Terry Collection have a relatively high degree of documentation (i.e., death certificates, anatomical preparation records) and contextual data (e.g., chronological age, sex, (social) race, occupation, CoD). Because of this, and like other historical documented collections (e.g., Hamann-Todd), the Terry Collection has been regularly, often uncritically, conceptualized and utilized as being representative of once living populations and as scientifically neutral^4,5,43,44^. However, individuals in the Terry Collection, like those in many other historical reference collections, represent a highly specific sub-population, with a distinct set of antemortem, perimortem, and postmortem experiences. Racial categorization on death certificates specifies that Black and/or African Americans comprise 54.4% of the Terry Collection, many of whom were of low socioeconomic status (e.g., “laborer”, typically construction, farm, domestic, or industry workers, as the listed occupation on death certificates)^45,46^ and arrived to Missouri from the Deep South as part of the Great Migration (c. 1910–1970)^4,40–42,45,47^. Black and/or African American individuals manifest higher rates of infectious disease, skeletal evidence of interpersonal violence, and skeletal and dental markers of cumulative physiological stress (e.g., periodontal disease), indicative of elevated local and systemic inflammatory burdens, than White and/or European American individuals in the collection^4,16,48,49^. These patterns represent embodiment of the harmful biological, social, and environmental conditions (e.g., crowded living conditions, poor sanitation, high rates of institutionalization) produced by 19th century enslavement, intergenerational effects, and harmful and insalubrious social, economic, biological, and environmental conditions in the Deep South as well as late 19th to mid-20th century St. Louis^41,47^. Most individuals in the Terry Collection, who died before 1955, also died in social relief institutions (e.g., public hospitals, asylums), primarily in St. Louis^40–42^. Afterwards, they were non-consensually dissected (anatomized) and subsequently incorporated into the Terry Collection^41^.

In the mid-19th to early-20th centuries dissection was highly stigmatized, viewed as punitive, and functioned as a form of on-going postmortem violence towards anatomized individuals and their communities^4,50^. Contemporaneous anatomy laws legalized dissection of unclaimed bodies from state-funded social relief institutions, which primarily served marginalized communities. This rendered individuals in these institutions vulnerable to dissection because they were not politically or economically able to resist it. Dissection and subsequent inclusion in documented collections both perpetuated and reinforced antemortem social inequality, acting as recurrent punishment for the deceased and marginalized communities, and reiterating forms of social control^4,44,50^. In this way, the Terry Collection and other historical reference collections represent products of structural violence and predominantly include individuals who experienced structural violence throughout their lives^4,44,50^.

## Results

### Synthesis of skeletal, dental, and systemic stress evidence

**Fig. 1:**
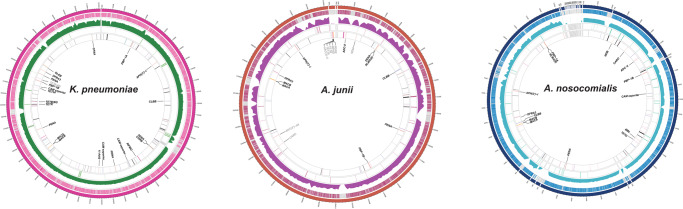
Pangenome analysis of St.LI reads mapping to the nearest reference genomes for *K. pneumoniae*, *A. junii*, and *A. nosocomialis*. The outermost ring shows genome coverage for each pathogen. Ring two shows gene presence (color) and absence (gray). Ring three shows the depth of coverage across the reference genome in 1 kb windows. Ring four shows resistance gene locations with presence (color) and absence (gray). Ring five shows antibiotic resistance genes by type (gray labels are absent) with select genes labeled.

Despite its rigid structure, the skeleton is a dynamic tissue that changes in response to human behavior and social, biological, and environmental conditions^51^. Among other factors, physical activity, diet and nutrition, experiences of trauma, and pathological processes, including chronic and/or episodic physiological stress, result in a range of hard tissue responses that, when assessed across the body, can be interpreted as a material archive of embodied conditions experienced over the life course^3,52,53^. Paleopathological skeletal and dental assessment of St.LI revealed musculoskeletal markers of physical activity, evidence of trauma, and infectious disease, alongside non-specific pathologies. St.LI exhibits enlarged and rugose postcranial muscle attachment sites and other musculoskeletal activity markers (Supplementary Data 1). They also exhibit a non-specific focal depression on the anterior aspect of the right femoral head and neck; remodeling (healing) periosteal reactions on multiple long bones; a remodeling, complete midline mandibular fracture; actively remodeling clustered pits and porosity on the scapulae and radii; and lytic pitting and porosity on the left femur, os coxae, right radius, and sternum, some with radiographic evidence of remodeling, while others were actively lytic at time of death, (Supplementary Data 2 and Supplementary Note 1 Figs. 1–4). St.LI experienced multiple oral pathologies, including dental caries, antemortem tooth loss (AMTL), oral abscesses, gingivitis, and periodontal disease (Supplementary Data 3 and Supplementary Note 1 Fig. 5). St.LI also exhibits several high sensitivity and specific indicators of tuberculosis (TB), following Dangvard Pedersen et al.^54^, as well as several less-specific indicators. This suggests that St.LI experienced chronic and/or episodic TB infection (Supplementary Data 4, Supplementary Note 1 Fig. 4, and Supplementary Note 1 Fig. 6). Furthermore, radiographically observable diffuse bone loss in the cranial vault, seen as well-remodeled areas of bone lysis (ectocranial) and proliferation (endocranial) on St.LI (Supplementary Note 1 Fig. 7), may also be associated with TB infection^7^.

The observed periodontal disease and probable TB infection likely impacted St.LI’s bodily stress response capabilities, potentially producing hyper-inflammatory and immunological suppression responses. This is because chronic and/or episodic physiological stress can heighten allostatic load (i.e., the cumulative burden of chronic stress), which can cause immunological suppression^55,56^, particularly of the cellular immune response against other pathogens^57^. The periosteal reactions and systemic porous lesions may be reflective of this process. Chronic local and systemic infections alter levels of systemic inflammatory response mechanisms, potentially dysregulating and elevating inflammatory cytokine expression^58,59^. This is seen during the active phase of TB infection—which St.LI was likely experiencing in the antemortem period—wherein cytokine levels (e.g., tumor necrosis factor alpha (TNFα)) are elevated^60^; these cells are also responsible for generating skeletal lesions^7^. This dynamic systemically affects the body via cascading immunological processes, since a hyper-inflammatory state can cause both localized and systemic inflammatory tissue damage and an elevated inflammatory response to other chronic infections, such as those causal to dental caries, periodontal disease, and potentially periosteal reactions^61,62^. Bi-directional relationships can also occur between oral pathologies and systemic inflammatory conditions; strong localized inflammatory responses, such as those in periodontal disease, can become systemic through spillover of inflammatory cytokines and other active immune cells. This can sometimes lead to chronic inflammatory systemic diseases^63^, although distinctive skeletal evidence of this process remains under study^62^.

### Oral metagenomic community profiles and pathogen identification

The ten most-abundant microbial species observed in St.LI’s dental calculus are known opportunistic or nosocomial pathogens (Supplementary Data 5). *Acinetobacter* bacteria, *A. junii* and *A. nosocomialis*, dominate (~48%) the recovered microbial community^64^ followed by *Klebsiella pneumoniae* as the third (~10%) most abundant bacterial species detected (Supplementary Data 5). Additionally, a competitive, reference-based mapping approach showed that over 669 times more reads mapped to the *K. pneumoniae* reference genome than to genomes of other potential causative agents. Any one or combination of these pathogens may be the causal organisms for St.LI’s CoD of lobar pneumonia (Table 1 and Supplementary Data 6). Overall, St.LI’s oral microbial community most closely resembles ancient tooth root communities and is distinct from soil and gut microbial profiles^65^ (Supplementary Note 1 Fig. 8b). Despite skeletal evidence suggestive of a TB infection, very few reads (0.004%) mapped to the *M. tuberculosis* reference genome^66^ (Supplementary Data 7); authentication with mapDamage (Supplementary Note 1 Fig. 9) and identification of the *M. tuberculosis* lineage was not possible.

**Table 1:** Percent of reads mapping to reference genomes of bacteria known to cause pneumonia.

Pathogen	Avg. read length (bp)	Percent of the total reads mapping to pathogen reference	Mean genome coverage
*Klebsiella pneumoniae*	67.8	9.262	11.155x
*Acinetobacter baumannii*	64.2	0.040	6.379x
*Staphylococcus aureus*	68.0	0.014	0.033x
*Coxiella burnetii*	49.5	0.012	0.029x
*Chlamydia psittaci*	45.7	0.003	0.011x
*Streptococcus pneumoniae*	38.0	0.002	0.003x
*Haemophilus influenzae*	40.1	0.002	0.004x
*Moraxella catarrhalis*	42.5	0.002	0.004x
*Neisseria meningitidis*	36.5	0.002	0.003x
*Chlamydophila pneumoniae*	43.5	0.002	0.008x
*Mycoplasma pneumoniae*	44.5	0.002	0.009x
*Legionella pneumophila*	42.1	0.001	0.001x
*Streptococcus pyogenes*	37.5	0.0003	0.0006x

Sorted by descending percent of reads mapping, bolded and boxed text indicates the highest mapping pathogen (see table S6 for references).

Following identification in the microbial community, pangenome analysis was performed for *K. pneumoniae*, *A. junii*, and *A. nosocomialis* (part of the *A. calcoaceticus*-*A. baumannii* complex), to determine similarities between the strains St.LI harbored and those in modern reference databases. For *K. pneumoniae*, the nearest match was identified as strain 12208 (13.8X, 91% genes shared, isolated from a sputum specimen), while for *A. junii* and *A. nosocomialis*, the nearest matches were strain WCHAJ59 (30.1X, ~81% genes shared, isolated from hospital sewage) and Ab22222 (28.4X, ~88% genes shared, isolated from scalp tissue), respectively (Supplementary Data 8). Reads mapping against the nearest reference genomes show relatively uniform coverage across most genomic regions (Fig. 1 and Supplementary Data 9). mapDamage analysis confirmed the presence of fragmentation and cytosine deamination patterns indicative of ancient DNA (Supplementary Note 1 Figs. 10–12).

Screening for virulence and antibiotic-resistance identified several genes associated with these functions in the strains found within St.LI (Supplementary Note 1 Figs. 13–18). The resistance genes found within St.LI are found in a large proportion of modern references. However, resistance genes associated with acute clinical strains are absent. The *K. pneumoniae* strain reconstructed in this study lacks the genes for yersiniabactin siderophore synthesis, found in the nearest modern reference. Yersiniabactin siderophore synthesis has been implicated in increased virulence^67,68^. The St.LI *K. pneumoniae* strain also shows orthologs for tetracycline-specific efflux pumps (*tetE*, *tetB*), aminoglycoside phosphotransferase (*aph(3’)-I*), beta-lactamase (*SHV-11*), and enterobactin biosynthesis (Supplementary Data 10 and Supplementary Note 1 Figs. 17, 18). In silico analysis determined the O-antigen and K-antigen types to be O1V2 and K54, respectively. Both St.LI *Acinetobacter* genomes harbor orthologs for macrolide efflux (*macAB*), ribosomal protection (*rlmA(II)*, *clbB*), trimethoprim resistance (*dfra3*), aminoglycoside phosphotransferase (*aph(3’)-I*), and common virulence-associated genes (*hemolysin*, *superoxide dismutase*, *macrophage infectivity potentiator*). The *A. junii* strain contains an adc-8 beta-lactamase, and chloramphenicol acetyltransferase (*cat*) (Supplementary Data 11 and Supplementary Note 1 Figs. 13, 14), whereas the *A. nosocomialis* strain has *adc-2* and *metallo-β-lactamases*, tetracycline-specific efflux pump (*tetD*), and a chloramphenicol exporter (Supplementary Data 12 and Supplementary Note 1 Figs. 15, 16). Bimodal SNP allele frequencies were observed for both *A. junii* and *A. nosocomialis*, suggesting the presence of one major strain at ~80% and a minor strain at ~20% abundance in St.LI’s metagenomic community (Supplementary Note 1 Figs. 10–12).

## Discussion

In addition to the acute infection responsible for their death, St.LI suffered from chronic and potentially episodic conditions (i.e., oral infections, probable TB), suggestive of immunological suppression. However, it remains unclear in what order St.LI developed these conditions and subsequent interactions of the involved immune response pathways. The healed mandibular fracture, activity markers, and diverse porous lesions and periosteal reactions (Supplementary Data 2 and Supplementary Data 3) indicate that St.LI experienced chronic and/or episodic physiological stress throughout late adolescence and/or early adulthood. St.LI’s stress responses were likely consistently active, suggesting that their allostatic load was exacerbated by the chronic oral and probable TB infections; dysregulated and hyperinflammatory immune responses may have resulted^61^. Further, established links between oral bacteria, and coronary and respiratory health risks indicate that immunological crosstalk between St.LI’s immune, circulatory, and respiratory systems may have occurred, creating opportunities for systemic comorbidities^63,69^ that are not skeletally identifiable, given current methods.

Molecular evidence of St.LI’s CoD from pneumonia corroborates the skeletal evidence of systemic disease. The most abundant taxa identified, *A. junii* and *A. nosocomialis* are strictly aerobic, recognized nosocomial and/or opportunistic microbes^70^. As oral abscesses are typically comprised of strict anaerobes^71,72^, the presence of *Acinetobacter* is likely associated with the active infection that potentially contributed to St.LI’s recorded lobar pneumonia CoD. The resemblance of this community to other tooth root microbial communities (Supplementary Note 1 Fig. 8) may reflect that calculus was sampled from an abscessed, first mandibular molar (Supplementary Note 1 Fig. 5 and Supplementary Note 1 Fig. 19). If so, the dental calculus reflects a depreciated microbial community and the continued, unhindered development of plaque despite the presence of an abscess (see Supplementary Note 1 for calculus formation discussion); oral microbes may have freely accessed nearby host circulatory and lymphatic vessels due to concurrent local inflammatory responses (i.e., periodontal disease). The simultaneous occurrence of the exposed circulatory and lymphatic vessels of the abscessed molar, and the continued deposition and calcification of dental plaque is potentially what made the incorporation of the *Klebsiella* and *Acinetobacter* bacteria possible from the concurrent infections (see Supplementary Note 1).

Several resistance genes commonly observed in modern clinical *Klebisella* and *Acinetobacter* isolates were detected in St.LI’s oral metagenome profile. Of particular interest are the genes conferring resistance to the beta-lactam class of antibiotics. While beta-lactam antibiotics are the first drug of choice for *Klebsiella* and *Acinetobacter* infections today, in the 1930s, these antibiotics were not widely used, as the first beta-lactam antibiotic, penicillin, was discovered in 1928^73^. Interestingly, the *SHV-11* (*K. pneumoniae*) and *ADC-8* (*A. nosocomialis*) genes detected in St.LI are identical to those observed in modern clinical isolates and are known to confer resistance to a diverse array of beta-lactam antibiotics including second- and third-generation drugs not commonly used until the 1960s^73–75^. The detection of these genes in St.LI highlights that antimicrobial resistance is a natural biological phenomenon that predates the recent, large-scale use of antibiotics^76,77^. In fact, *Klebisella* and *Acinetobacter* species are hypothesized to be a natural reservoir for *SHV* and *ADC* class beta-lactamases, respectively^74,75^. Overall, St.LI’s atypical oral microbial community, and its enrichment with opportunistic (commonly nosocomial) pathogens, combined with the skeletal lesions, suggest St.LI experienced an incapacitated immune system and subsequent elevated susceptibility to coinfections.

In conjunction with the high morbidity associated with any *K. pneumoniae* infection, the chronic oral infections, probable TB infection, and the *Acinetobacter* pathogens would have likely suppressed St.LI’s ability to effectively combat pneumatic infection, regardless of his young age^78^. *K. pneumoniae* infections progress and present rapidly^79^; if St.LI did not or was not able to seek medical attention and specific, effective treatment(s)^80,81^, his condition would have quickly deteriorated. St.LI’s pneumonia would have resulted in his lungs filling with fluid, ultimately causing death through suffocation at the end of the recorded 11-day hospital stay.

The concept of embodiment, coupled with ecosocial theory, makes broader synthesis across the presented data possible. Embodiment holds that the human body physiologically incorporates their social, environmental, and biological conditions^44,82^. Ecosocial theory proposes that humans embody exposures from socially patterned, exposure-induced, disease-causing pathways, which are mediated by biological processes (e.g., gene expression, physiology); this explains individual health outcomes and, when individuals are aggregated, population-level health inequities^83–85^. Social race (e.g., Black, White), like other modes of social inequality, is a culturally constructed framework of structural advantage and disadvantage with both social and biological consequences (e.g., access to medical resources, exposure to stressors)^86^. Therefore, health inequities (i.e., reduced longevity) associated with race represent embodied inequality, which arises through embodiment of structural-level (e.g., social closure) and individual-level (e.g., interpersonal discrimination, root shock) race-based discrimination^86,87^. Information from death certificate data, disease pathogenesis, and immunopathologies, as well as skeletal and microbial data provide insight into aspects of St.LI’s lived experiences as a young, Black and/or African American laborer and  the embodiment of racial discrimination during a period of great social division and change in St. Louis and the broader US. The evidence of trauma and chronic physiological stress evident in St.LI’s microbiome and skeleton, and the skeletons of other Terry Collection individuals, can be associated with the national-level processes of race-based structural violence and racialized terror in the late 19th to mid-20th century Jim Crow-era US, and local, St. Louis-level processes of social closure, anti-Black political violence, and White power politics. St.LI’s life was likely socially, mentally, and physiologically stressful. For example, in the absence of patient-specific documentary evidence we cannot be certain, but St.LI’s mandibular fracture may be associated with discriminatory practices; Southern-born Black and/or African Americans, like St.LI, were commonly used as strike-breaking laborers in the midst of wide-scale labor unrest, and economic strain and upheaval affecting industry in St. Louis^16^. More broadly, the evidence of chronic physiological stress and systemic and local infection evident throughout St.LI’s skeleton may represent embodiment of diverse inequalities experienced by St. Louis’s Black and/or African American communities^48^, such as St. Louis’ residential segregation ordinance, the 1917 East St. Louis race massacre, crowded living conditions, poor sanitation, and inadequate access to and poor quality health care^27,41,45^. City Hospital #2, which St.LI died in, was especially overcrowded and unsanitary throughout the 1920s and 1930s^38,88^. These conditions resulted in poor patient health outcomes^34^, such as those experienced by St.LI, and contributed to and exacerbated health disparities^33,36^ in St. Louis.

St.LI and his community were likely further impacted by national-level structural processes and events, and their localized expressions, including the 1909 establishment of the National Association of the Advancement of Colored People (NAACP) in response to violence and discrimination; federally mandated segregation in 1913; the intensification of the Great Migration, which carried many Black and/or African Americans to St. Louis; the 1921 Tulsa race massacre; Jim Crow laws; the Great Depression; the Harlem Renaissance; Prohibition^89^; and World War I. Together, these national- and local-level processes and events represent the deep, historical discriminatory roots of health inequities within BIPOC communities in the present-day^16,41,45^.

The increasingly personalized information and narratives obtainable from deceased individuals via modern, interdisciplinary techniques that are progressively available have revealed previously invisible aspects of St.LI’s ante- and perimortem experiences, including information about his systemic health status and the public hospital conditions that St.LI experienced. While for some communities, portraits like this may represent valuable remembrances and opportunities to tell untold histories, this view may not be universal. It is well documented that because of the collection processes involved in their construction and the individuals targeted for inclusion within them, some historic documented reference collections, such as the Terry Collection, are products of structural violence^44,90^.

Accordingly, research involving reference collections must consider the ethical issues surrounding many of these collections^91^. Reference collections (i.e., anatomical, medical) have been fundamental to the development of methods and procedures in anthropology and medicine, such as the development of sex, age, and ancestry estimation methods, and diagnostic criteria for pathological conditions^40,92^. But researchers in the medical and academic communities who are conducting molecular analyses with historical reference collections, which may include individuals with living descendants, must prioritize the protection of personally identifiable information and of anatomical personhood, especially in the age of genetic testing and interest in genealogy. This may include, but is not limited to anonymizing individuals, not analyzing human genomic data (unless requested by descendant(s) and/or communities), and recognizing individuals within these collections as research participants^4^, as we do here. Active recognition of the social history of historical reference collections, and the embodied lived experiences of included individuals, as well as how information gained from these collections may affect living communities, should be evaluated during project design to not further perpetuate the structural violence inherent to many historical reference collections and scientific research performed on them^4,6,43,44^. In doing so, the temporal and ideological gaps between researchers and the analyzed individuals can be bridged, literally parlaying how the past informs the present.

Normalizing the identification and discussion of social inequalities deriving from racist paradigms is critical for accessible and meaningful scientific knowledge production; a driving question that needs to be continually asked throughout the research process is: who benefits from the scientific knowledge being generated^93^? This is especially important when working with deceased individuals because the individuals under study can no longer directly benefit from the information they reveal (see Study Transparency Statement). Indeed, there is increasing momentum across multiple museums in the US, including the Smithsonian Institution’s National Museum of Natural History which houses the nation’s largest collection of human remains, including the Terry Collection, to develop guidance on the study of Black and/or African American, and marginalized American individuals in their collections^94,95^. While not a new concept^93,96^, scientific research must position itself to be more reflective of how generated information affects living communities and represents the past.

By considering multiple lines of evidence, the present study reveals a great breadth and depth of otherwise inscrutable information about the antemortem and perimortem experiences of St.LI. This study also reveals that dental calculus, once thought to be a cumulative substrate, can also incorporate evidence of acute infections, potentially making numerous, otherwise invisible, health and disease conditions accessible in the bioarchaeological record. To the best of our knowledge, this is the first time *K. pneumoniae* and multiple *Acinetobacter* genomes have been reconstructed from human skeletal remains and identified as potentially direct contributors to CoD. This interdisciplinary evidence culminates to depict a young, immunocompromised individual whose poor health and death experiences embodied the inequalities experienced by many Black and/or African American, and other historically marginalized communities in the late 19th and early 20th centuries. Critically, embodied inequality continues to be reflected as disproportionate morbidity and mortality from respiratory infections, including COVID-19, in contemporary BIPOC communities. The structural violence that contributed to St.LI’s health outcomes in 1930s St. Louis continue to drive health outcomes in many communities today^97^.

## Materials and methods

### Skeletal analysis

Skeletal and paleopathological inventories were performed following established standards^3,7,54,98,99^. These included macroscopic assessment of oral health and pathological lesions such as those associated with infectious diseases (e.g., TB). Radiographs of femora and cranial vault pathologies were taken to visualize the underlying trabecular structure and the extent of bone remodeling.

### Sampling, DNA extraction, library preparation, and Illumina shotgun sequencing

Subsampling of dental calculus was conducted at the National Museum of Natural History’s (NMNH) Museum Support Center (Suitland, Maryland). In accordance with previous studies^12^, a bleach sterilized dental scalar and nitrile gloves were used to scrape dental calculus directly from the individual into a sterile 1.5 ml tube.

All laboratory work was performed at the Laboratories for Molecular Anthropology and Microbiome Research (LMAMR) at the University of Oklahoma (Norman). Dental calculus was processed for DNA extraction and library preparation in the LMAMR Ancient DNA Laboratory, a dedicated, six-chambered clean room, in accordance with established contamination control workflows, including physical separation from other laboratories performing molecular research, unidirectional work, and positive air pressure, to avoid contamination. A full-body Tyvek suit, hairnet, facemask, and double gloves were worn to protect the sample from contamination.

DNA extraction followed previously established protocols with a slight modification^100^: the sample was not UV-irradiated prior to the EDTA wash. UV-irradiation was not applied so as to have as much of the dental calculus as possible entering the extraction; the dry/original dental calculus sample was very small, flakey, crumbly, and static, causing it to be easily lost when sample tubes were opened or exposed to the positive pressure air flow of ancient DNA laboratories. Briefly, 4.9 mg of dental calculus from St.LI’s right, mandibular first molar was washed with 0.5 M EDTA for 15 min, followed by decalcification in a solution of (fresh) 0.5 M EDTA and 10% proteinase K (Qiagen) at room temperature on a nutator for 72 h. A modified Qiagen MinElute silica-column based purification protocol was used, with final elution in a 60 µL volume (Buffer EB, Qiagen). The eluate was then quantified using the dsDNA High Sensitivity assay on the Qubit fluorometer (Life Technologies), yielding 0.176 ng/µL DNA (2.15 ng DNA per mg of dental calculus).

Partial uracil-DNA-glycosylase (UDG) treatment was performed to preserve terminal nucleotide damage for later authentication of the DNA sequences as “ancient”^101^. DNA extract (30 µL) was treated with partial UDG and built into a dual-indexed library using the NEBNext DNA Library Prep Master Set (E6070), following the manufacturer’s instructions, with the exception of the nebulization step to not further fragment the DNA, as well as minor modifications. Partial UDG treatment was followed by end repair using the NEBNext End Repair enzyme and a 30-min incubation at 20 °C. Other end repair buffers were not added because reaction reagents were already present from the partial UDG treatment. The end-repaired product was purified using the MinElute PCR Purification kit (Qiagen) with the manufacturer’s protocol and eluted in 15 µL EB. Illumina-compatible adapters were ligated using the NEBNext Quick Ligation Buffer and Quick T4 Ligase in a 15-min incubation at 20˚C, followed by another MinElute purification (15 µL EB elution). An adapter fill-in step was performed using the NEBNext Reaction Buffer and Bst Polymerase with incubation at 37 °C for 20 min, followed by a final MinElute purification in a volume of 50 µL. The library was quantified with quantitative PCR assay (qPCR; Roche). Dual-indexing was performed using the Kapa Hifi Uracil + kit (12.5 µL master mix, 1 µL of 2.5 mg/ml BSA, 0.75 µL of 10 µM forward and reverse primers containing unique barcodes, and 4 µL library, in a final 25 µL reaction volume). Thermocycling conditions were 5 min at 98˚C followed by 14 cycles of 98 °C (20 s), 60 °C (15 s), and 72 °C (30 s), followed by a final elongation at 72 °C for 1 min. Indexing was performed in triplicate; amplified products were pooled, and purified using MinElute purification and eluted in 30 µL. Extraction and library controls were processed simultaneously along with the sample.

The purified library was analyzed using the Fragment Analyzer (Agilent) and pooled at equimolar ratio along with other project samples. The Pippin Prep (Sage Biosystems) was used to remove adapter dimers by performing a size-selection at a target range of 150–500 bp. The pooled libraries were sent for sequencing on an Illumina NextSeq (2 × 75 bp) at the Max Planck Institute for the Science of Human History (Jena, Germany). We have deposited sequencing data, sans reads mapping to the human genome, at the NCBI Sequence Read Archive (http://www.ncbi.nlm.nih.gov/sra) under the BioProject ID PRJNA851947.

### Oral metagenomic data processing and authentication

Raw reads were quality-filtered before downstream analysis. Adapters were removed, paired-end reads were merged and quality-filtered for base quality (Q20), read length (30 bp), and ambiguous bases (‘N’), using AdapterRemoval (v2.1.7)^102^. The sample had 9,052,598 analysis-ready reads; blanks were sequenced to a depth of 0.7–8.0 million analysis-ready reads.

To determine the endogenous human DNA content, analysis-ready reads were mapped to the human reference genome (hg19) using Bowtie2 (v 2.3.4.1)^103^ with default parameters and the --no-unal option. SAMTools (v1.5)^104^ was used to filter out mapped reads with Phred quality less than 30 and remove duplicates using *rmdup*. The endogenous human DNA content of the St.LI library was 1.3%, as compared to less than 0.34% for the blanks (Supplementary Data 13). To authenticate the reads as ancient, mapDamage (v2.0.8–2)^105^ was used to estimate the nick frequency, cytosine deamination rates, and fragment length distribution, which were then visualized in R using ggplot2^106^ (Supplementary Note 1 Fig. 8a). The recovered human DNA fragments had short read lengths (62 bp average ± 23.2) and characteristic ancient DNA terminal base damage. Further analysis on recovered human DNA was not conducted to protect the genetic confidentiality of St.LI and possible descendants. Beyond read authentication, we did not conduct further analyses (i.e., genetic ancestry) on sequences mapping to the human genome (hg19), as this was outside the original study parameters and due to the lack of consent from potential descendants (lineal or communal). Human DNA sequences have been removed from the available SRA data.

Analysis-ready reads were mapped to the GreenGenes database (97% pre-clustered, v13.08)^107^ using Bowtie2 with default parameters, followed by removal of PCR duplicates using SAMTools *rmdup*. Unique reads mapping to the Greengenes database were extracted and used as input for closed reference OTU-picking in QIIME^107^ (v1.9, uclust algorithm, 97% pre-clustered Greengenes database, max_accepts = 500, max_rejects = 500, word_length = 12, stepwords = 20, enable_rev_strand_match = True). For the blanks, only 123–1605 unique reads mapped to the GreenGenes database, and hence, the blanks did not pass the threshold of 4000 reads used for rarefaction (table S14). Genus level and species level taxonomic summaries were generated using QIIME and used as input for SourceTracker (v1.0.1)^108^ to assess the contribution of various oral and non-oral sources, including modern human skin, supra- and sub-gingival plaque, soil, and ancient dental calculus and tooth root samples^65^. Terry Collection individuals were never buried^40^.

### Species profiles and pangenome analysis

Analysis-ready reads were processed using MetaPhlAn3^109^ to generate species-resolved community profiles (table S5). The dominant organisms identified include *Acinetobacter junii* (~27%), *Acinetobacter nosocomialis* (~22%), and *Klebsiella pneumoniae* (~10%). To determine the gene content of the strains observed in St.LI, a pan-genome analysis was performed. Complete reference genomes were obtained from the NCBI RefSeq database (August 2019; *A. junii*, *n* = 57; *A. nosocomialis*, *n* = 98; and *K. pneumoniae*, n = 391) and pan-genome databases were constructed using PanPhlAn3^110^ with default parameters. Analysis-ready reads were mapped to this pan-genome reference with default parameters, and a gene presence-absence table was generated using PanPhlAn3. Additionally, the pan-genome reference catalogue was screened for putative virulence and antibiotic resistance genes using the Virulence Factor database (VFDB R5, May 2020)^111^, and DeepARG (October 2019)^112^ respectively (Supplementary Data 9–11).

Analysis-ready reads were mapped to the nearest reference genomes (identified from PanPhlAn3) as well as type strains for *A. junii*, *A. nosocomialis*, and *K. pneumoniae*, respectively, using BWA (v0.7.12)^113^ with the parameters -l 1024, -n 0.03, -q 37 as suggested for ancient DNA (Supplementary Data 8)^114^. The presence of *Mycobacterium tuberculosis* (TB) was also investigated by mapping reads to the reconstructed ancestral *M. tuberculosis* reference genome^66^. PCR duplicates were removed using DeDup^115^ and mapDamage was used to assess DNA damage patterns. For type strains, variant-calling was performed using SAMTools mpileup and VarScan (v2)^116^ and the following parameters: --min-coverage 5, --min-reads2 3, --min-avg-qual 30, --min-var-freq 0.2, --min-freq-for-hom 0.9, --p-value 1, --strand-filter 0.

### Study transparency statement

This study was originally conceptualized as a part of a larger methodological project designed to test the potential of dental calculus for recovering biomolecular evidence of acute infectious diseases from bioarchaeological individuals. The Terry Collection was selected because of the number of individuals with clinical, antemortem CoD from infectious diseases. However, early in the project, the study shifted to focus on St.LI’s individualized narrative, the potential of biomolecular data from dental calculus to provide direct evidence of embodied inequality and structural violence, and scientific policies for ethical research involving historical reference collections. Ethical scholarship within bioarchaeology entails attention not only to the origins of the studied individuals, but also the contextual factors that resulted in their amassment^16^. As part of this redirection, we have engaged with Smithsonian Institution leaders, about the significance of the personalized history presented here and how future research could be impacted and ethically informed; current Smithsonian Institution policy holds that individuals in the Terry Collection are de-identified, meaning that members of the descendant community for individuals in the Collection cannot currently be identified. At the time of writing, several authors continue to be a part of negotiations surround institutional responsibilities and generating guidelines for research and curation surrounding the Terry Collection and historical documented collections more broadly.

St.LI was not and could not be informed of his rights and roles in the current study, nor any of the previous research performed on the Terry Collection since its establishment. St.LI, alongside other individuals included in the collection before 1955, were not able or contemporaneously permitted to provide written, informed consent and did not have the choice to withdraw from the study or inclusion in the collection; ethical research mechanisms are designed to protect research participants but cannot be retroactively applied to deceased individuals. This ethical paradox highlights the lack of consideration for the universal rights of deceased individuals, especially of members of historically marginalized communities and social identities, within much of anthropological research. Due to the personal history and information synthesized in this study, we recognize St.LI as an active participant in this research, following de la Cova^4^, which explicitly recognizes and acknowledges their personhood; much of this study is meant to bring some of St.LI’s life history, previously obscured and erased, into broader recognition, fostering a shared remembrance of St.LI and other individuals in the Terry Collection with similar experiences, while recognizing their personhood.

The authors recognize the complexity of attempting this goal while simultaneously anonymizing St.LI. Here, we have chosen not to disseminate human genetic and other personalizing information for St.LI due to the history of medical exploitation^27^ and the increasing impact of genetic ancestry studies and services on society (e.g., appropriation of genetic ancestry results to claim Black and/or African American and Indigenous community identity, scholarship, and culture in the United States^117–120^), attempting to minimize further disruption to the rest and privacy of St.LI and his possible descendants. More broadly however, we argue that it is imperative that curatorial institutions and wider research communities utilizing historical documented collections develop decolonized research, publication, training, and curation guidelines and policies that actively recognize the personhood and embodied life histories of the individuals included in documented collections^96,121^. These policies should further translate into repatriation for individuals accompanied by individual-specific documentation and identifiable descendant(s) and/or communities. Repatriation need not be conceptualized as the loss of scientific resources, but rather as an imperative pathway for establishing integral, meaningful, long-term relationships with descendant communities, and which could enable community engaged and directed research^96,121^. Community informed, engaged, and directed research also carries the benefit of ensuring wider public interest in the generated knowledge, and the direct applicability and utility of this knowledge for people and communities involved.

Importantly, through research and curation practices that re-establish personhood for individuals in historical documented collections, the presumed scientific neutrality of the collection and research is stripped. Inclusion in historical documented collections, and many of the research projects they have been incorporated into, have deeply dehumanized the included individuals; ignoring whole individuals and their embodied life histories, for instance, to instead prioritize curation and analyses of single skeletal parts (e.g., crania), rendering individuals into scientific objects^4,43,121^. A recognized practice for re-establishing personhood (i.e., rehumanizing) and positioning individuals in historical reference collections as subjects is osteobiographical or case study-based research focused on reconstructing the embodied life history experiences of individuals within their specific cultural and temporal context^4,15,16^, such as we employ here. We have tried to contextualize the osteological, metagenomic, and skeletal evidence within Black and/or African American socio-political history in St. Louis and the US in the early 1900s.

Enacting decolonized and community-based research practices is not new but has yet to become standard practice. Indeed, landmark projects like the New York African American Burial Ground and the First African Baptist Church^122,123^ provide established frameworks and models for ethical curation, research, training, and community outreach and education practices. These enable the re-establishment of personhood and recognizes the socially constructed nature of scientific research and results. These frameworks shift the authority and agency to the historically oppressed and marginalized descendent communities, positioning them as the ethical clients for a given project (i.e., clientage model)^121,122^ and represent an empirical, ethical, and social justice-based pathway for anthropological research going forward.

### Reporting summary

Further information on research design is available in the Nature Research Reporting Summary linked to this article.

## Supplementary information

Supplementary Information

Description of Additional Supplementary Files

Supplementary Data 1-14

Reporting Summary

## Data Availability

The NGS sequencing data that support the findings of this study have been archived in NCBI Sequence Read Archive (SRA) under the BioProject ID PRJNA851947. Human DNA sequences have been removed from the available SRA data to protect the genetic identity and potential genealogical descendants of St.LI. The data used for pathogen analyses are available in Supplementary Data 10–12.
