# Publisher Correction: Remembering St. Louis individual—structural violence and acute bacterial infections in a historical anatomical collection

**DOI:** 10.1038/s42003-022-04063-8

**Published:** 2022-10-31

**Authors:** Rita M. Austin, Molly Zuckerman, Tanvi P. Honap, Hedwig Lee, Geoff K. Ward, Christina Warinner, Krithivasan Sankaranarayanan, Courtney A. Hofman

**Affiliations:** 1 University of Oklahoma, Laboratories of Molecular Anthropology and Microbiome Research, Norman, OK 73019 USA; 2 University of Oklahoma, Department of Anthropology, Norman, OK 73019 USA; 3 Smithsonian Institution, National Museum of Natural History, Department of Anthropology, Washington, DC 20560 USA; 4 Mississippi State University, Department of Anthropology and Middle Eastern Cultures, Mississippi State, MS 39762 USA; 5 Washington University in St. Louis, Department of Sociology, St. Louis, MO 63130 USA; 6 Duke University, Department of Sociology, Durham, NC 27708 USA; 7 Washington University in St. Louis, African and African-American Studies, St. Louis, MO 63130 USA; 8 Harvard University, Department of Anthropology, Cambridge, MA 02138 USA; 9 University of Oklahoma, Department of Microbiology and Plant Biology, Norman, OK 73019 USA; 10 Present Address: Natural History Museum, University of Oslo, Oslo, 0318 Norway

**Keywords:** Bacterial infection, Bacterial genetics, Ethics, Microbiome, History

Correction to: *Communications Biology* 10.1038/s42003-022-03890-z, published online 03 October 2022.

In this article the affiliation details for Geoff K. Ward were incorrectly given as ‘Duke University, Department of Sociology, Durham, NC 27708, USA and Washington University in St. Louis, African and African-American Studies, St. Louis, MO 63130, USA’ but should have been ‘Washington University in St. Louis, African and African-American Studies, St. Louis, MO 63130, USA’. The original article has been corrected.

