# Supplementary material for: Remembering St. Louis Individual—structural violence and acute bacterial infections in a historical anatomical collection
Source: Commun Biol. 2022 Oct 3;5:1050. doi: 10.1038/s42003-022-03890-z (PMC9527723; doi:10.1038/s42003-022-03890-z)
Supplement: Supplementary file 1 — Supplementary Information [file 42003_2022_3890_MOESM1_ESM.pdf]

Supplementary Note 1

Remembering St. Louis Individual—Structural Violence and Acute Bacterial Infections in a Historical Anatomical Collection

Rita M. Austin*, Molly Zuckerman, Tanvi P. Honap, Hedwig Lee, Geoff K. Ward, Christina Warinner, Krithivasan Sankaranarayanan, Courtney A. Hofman*

*Corresponding authors. Email: austinrmca@gmail.com and courtney.hofman@ou.edu

<u>Dental Calculus Formation</u>

The amount and rapidity of calculus formation is influenced by diet, genetic variation, oral hygiene practices, age, sex, and mineral levels (calcium and phosphate) of the blood and saliva[1,2]. While dental calculus may form within 2-week periods[3], there is considerable variation between individuals, with people being described as "heavy" and "light" calculus formers. Several studies estimate that 0.10-0.15% of calculus by dry mass is deposited, daily[4]. While we cannot assess whether St.LI exhibited "heavy" or "light" calculus deposition, the unusual microbial composition indicates the consistent exposure of the mouth and dental calculus to *Acinetobacter* and *Klebsiella* bacteria. The reconstructed oral community of St.LI clustering more closely with tooth roots than dental calculus samples further emphasizes St.LI's dysbiotic oral health and may reflect the exposure of the mouth and dental calculus to *Acinetobacter* and *Klebsiella* bacteria. The presence of pathogens associated with acute infection immediately preceding death suggests that dental calculus is not just a long-term reservoir of microbial activity but may also be a source of transitory and acute biological organisms and conditions.

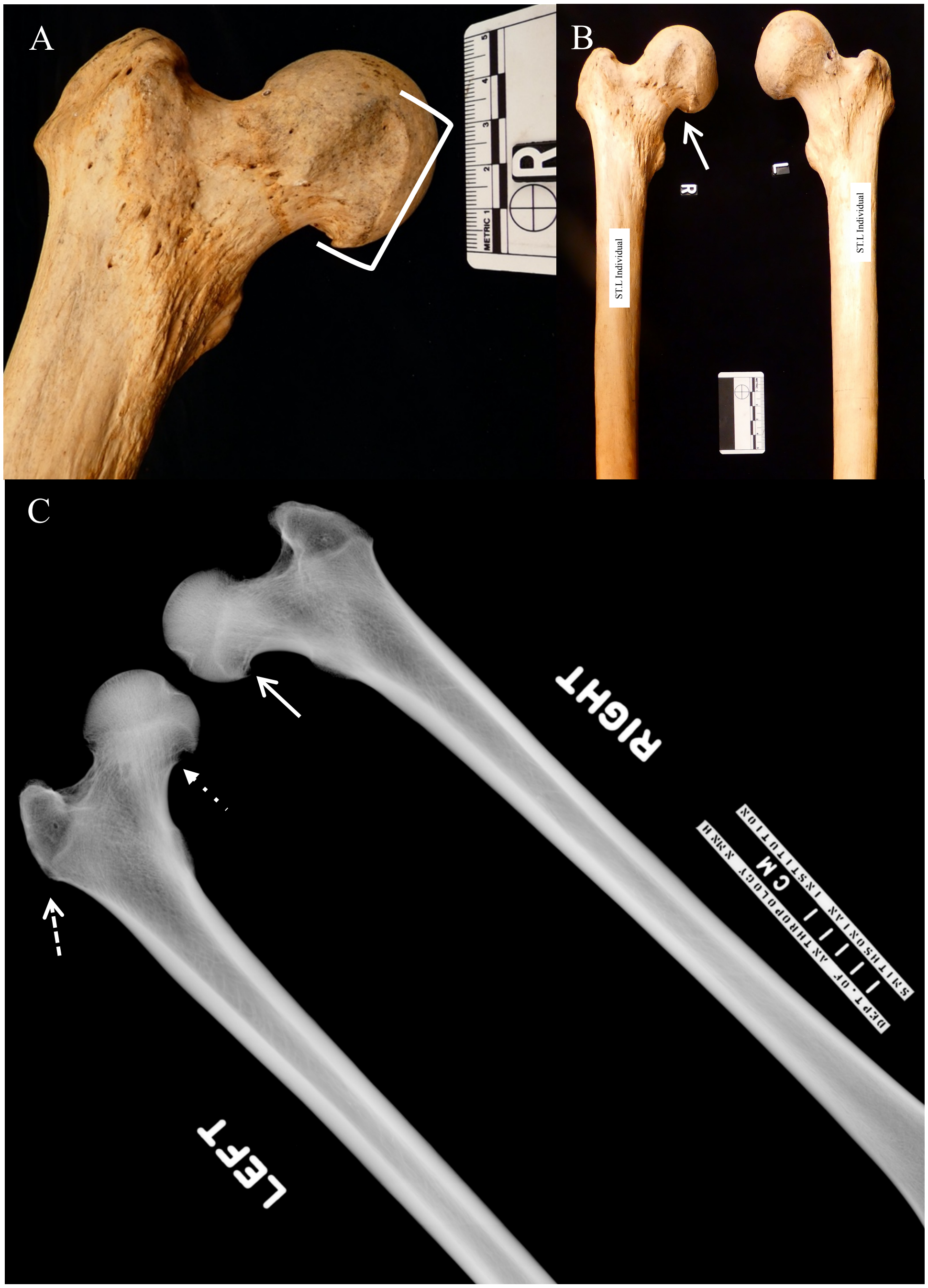

Supplementary Fig. 1 Femur focal depression photos and radiograph.

(A) Focal depression with clear margins on inferolateral portion of the anterior aspect of right femoral head, extending onto the anterior portion of the femoral neck (c. 23 mm in maximum diameter). The depression exhibits rounded margins, indicating a long-standing presence, with minimal evidence of macroscopic and radiographic remodeling of the cortical and trabecular bone. A review of the published literature has not yielded the etiology of this depression (e.g., activity related). (B) The trait (arrow) is not bilaterally symmetrical. (C) Radiographs of the left and right femora also show limited reorganization of the trabecular bone underlying the depression on the right femur (solid arrow). The left femoral head also shows osteolytic bone

resorption occurring on the inferior aspect (dotted arrow) and a lytic lesion within the trabecular bone (dashed arrow), that is not observable on the bone cortex.

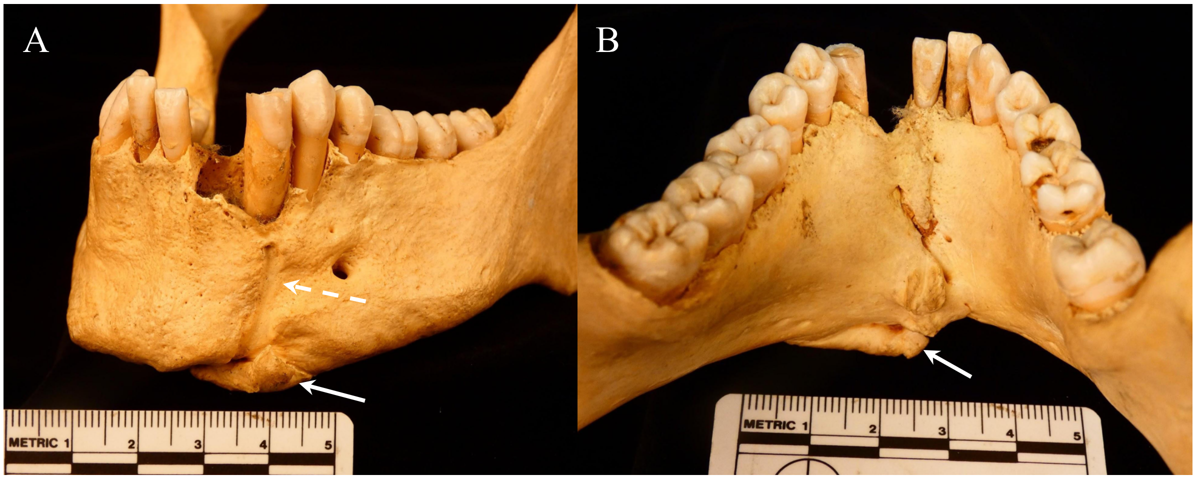

Supplementary Fig. 2 Mandibular mid-line fracture.

(A) Anterior and (B) posterior views of complete midline mandible fracture. The calcified hematoma (solid arrows) and probable fracture line (dashed arrow) are indicated.

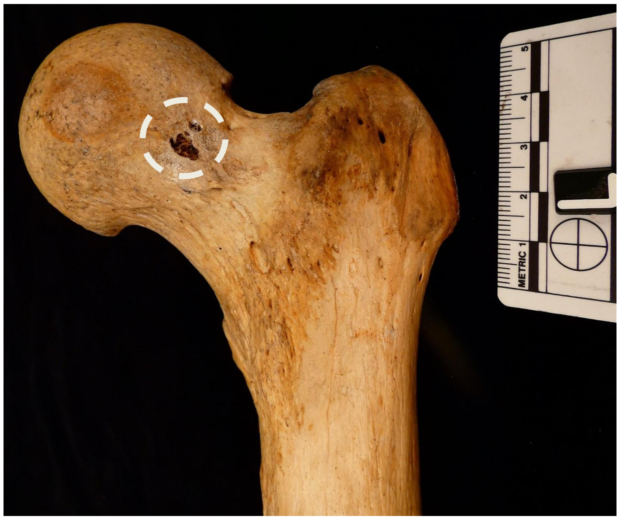

Supplementary Fig. 3 Lytic pitting of proximal femur.

Anterior view of the proximal left femur showing two clustered lytic pits (inferior pit: c. 23 mm in maximum diameter; superior pit: c. 10 mm in maximum diameter). The margins are sharp, meaning that the lesion was likely active at time of death, with irregular trabecular bone within the base of the pits.

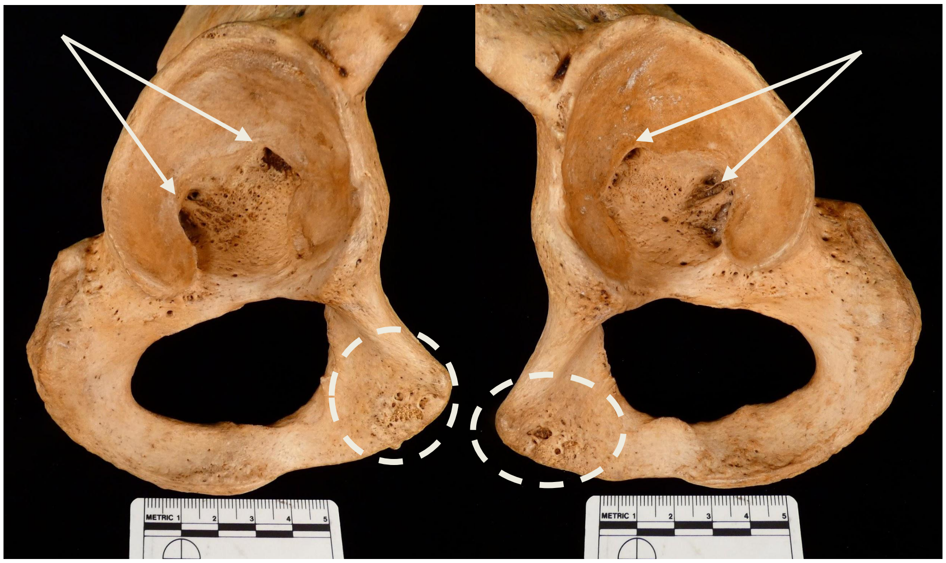

Supplementary Fig. 4 Reactive bone of the os coxae.
The right (A) and left (B) os coxae. Clustered pits, with rounded margins indicative of healing, are observable on medial portion of the pubic crest (dashed circles). The arrows indicate the presence of multiple deep cavities with lytic pitting in the acetabulae. The borders of the pits exhibit woven bone growth with dense trabeculae. The lesions on the right (A) os coxa have sharp margins, indicating that they were likely active at time of death, while those on the left (B) have rounded margins, which is indicative of healing. These lesions correspond to the ACE2 indicator, which has high diagnostic value, for tuberculosis infection[5].

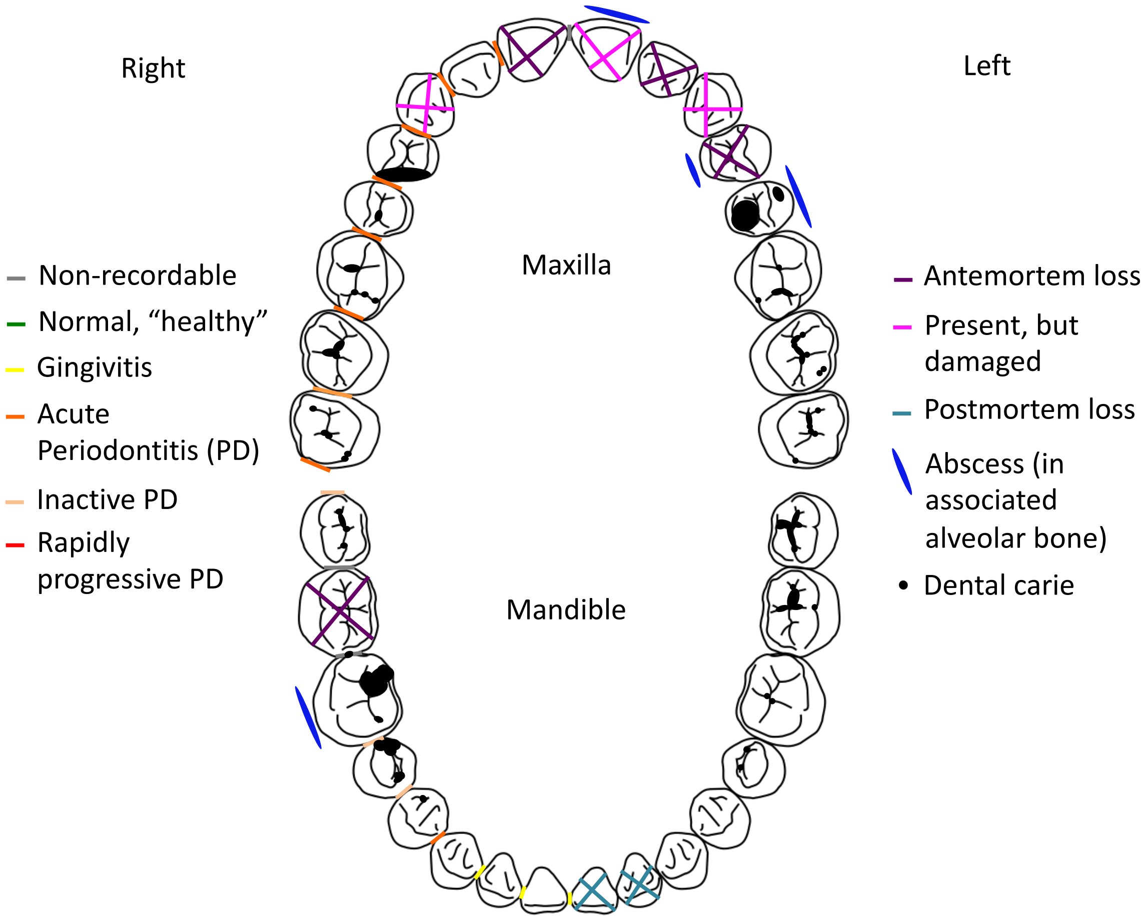

Supplementary Fig. 5 Dental disease schematic.
Schematic indicating tooth and alveolar bone location of dental caries, antemortem and postmortem tooth loss, periodontal disease, gingivitis, and oral abscesses.

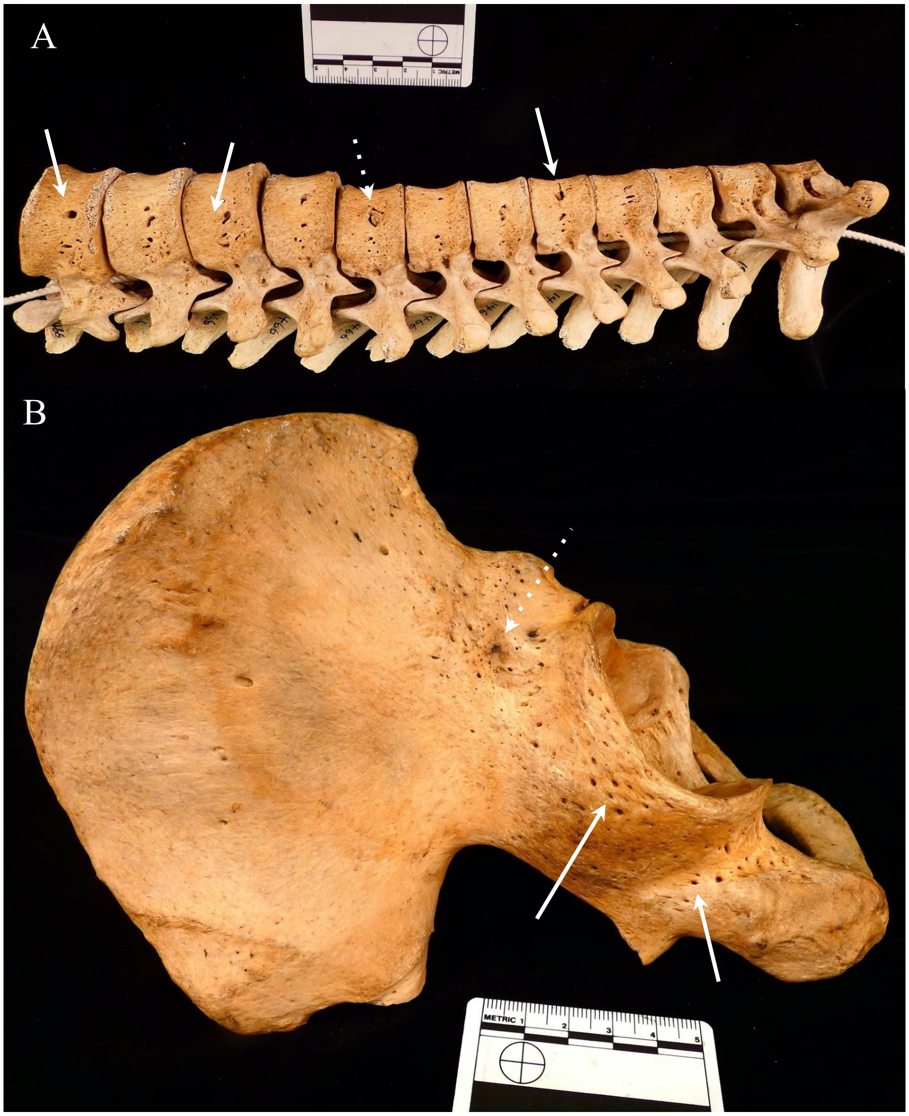

Supplementary Fig. 6 High diagnostic value tuberculosis indicators.
(A) Several vertebrae exhibit large pores or pits (solid arrows). Several thoracic vertebrae, specifically T1 to T12, have irregular to circular shaped pits (measuring ≥3 mm in maximum diameter) with rounded margins, indicative of healing. Several lumbar vertebrae also have clusters (≥3) of large pits with irregular to circular shapes and rounded margins, specifically L1 to L5. Several of these have irregular trabecular bone at the base (dotted arrow) and correspond to the VEN1 indicator for tuberculosis lesions[5]. (B) Multiple large, roughly circular pits, are present on the right os coxae, with several measuring ≥3 mm in diameter. These lesions correspond to the BOD indicator for tuberculosis[5], which has high diagnostic value. Most have rounded margins, indicative of healing (solid arrows), though one lytic foci has sharp margins, and was likely active at time of death (dotted arrow).

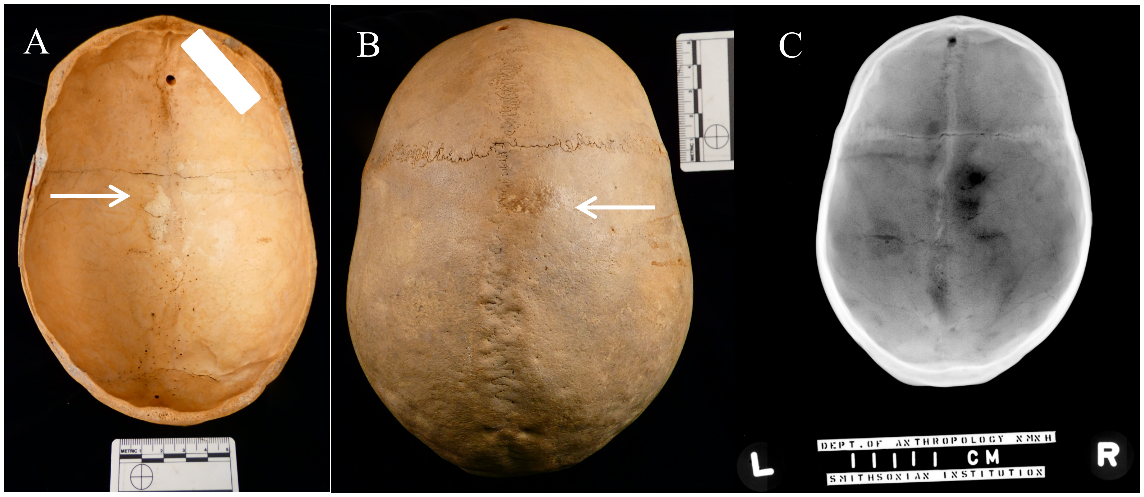

Supplementary Fig. 7 Cranial vault photos and radiograph of pathological lesions. The cranial vault displays several pathological lesions, which may be associated with Tuberculosis. (A) The endocranial surface exhibits an area of well-remodeled, healing reactive bone located along the mid-point of the sagittal suture (white arrow). Deidentification made with white box. (B) The ectocranial surface shows an area of diffuse, well-remodeled, healing lytic destruction located along the mid-point of the sagittal suture (white arrow). (C) On the radiograph of the cranial vault, diffuse bone loss (radiolucent areas) is evident in several areas of the cranial vault, alongside the sagittal suture and on the parietal bosses. The largest area of loss is indicated (white arrow).

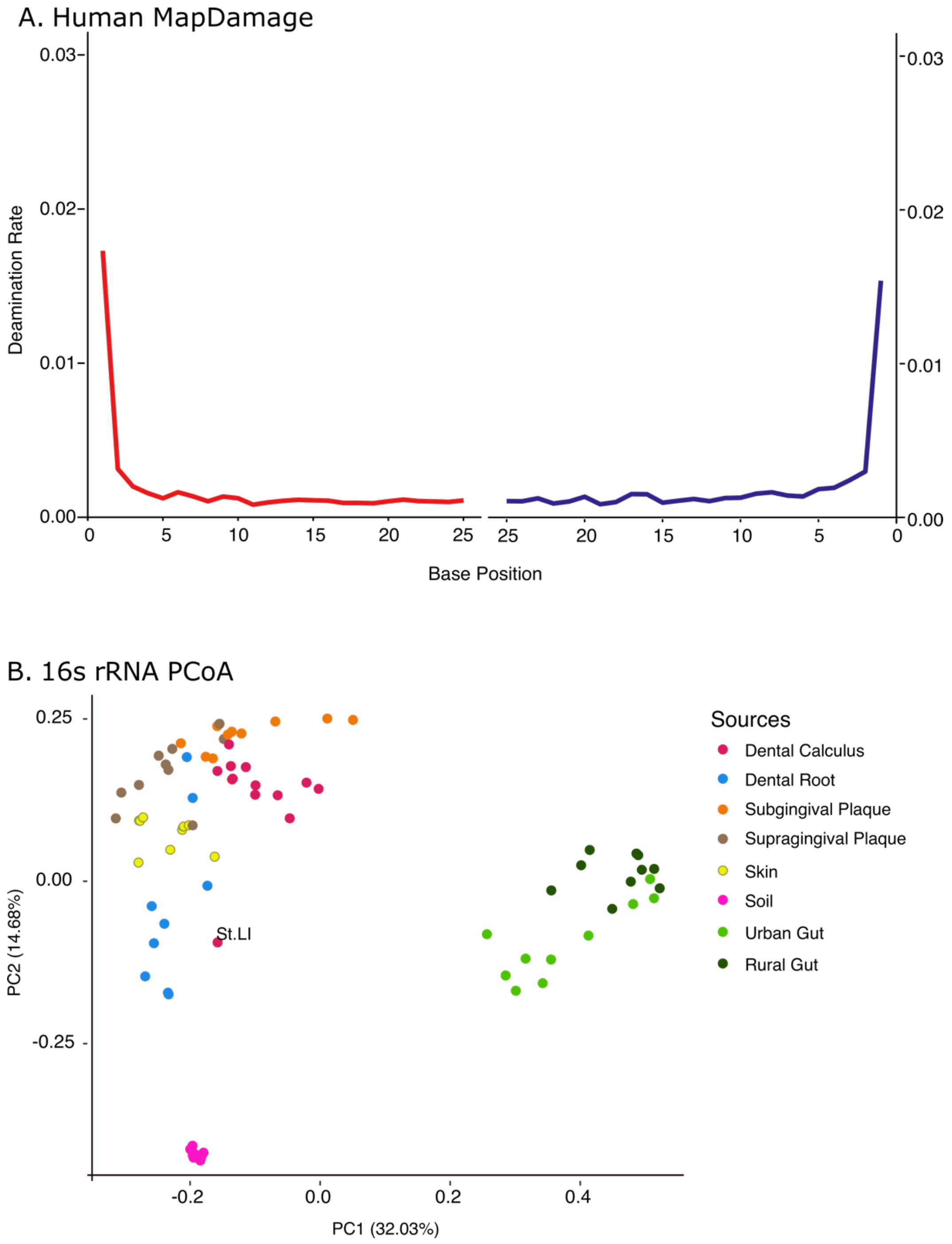

Supplementary Fig. 8 Authentication of Dental Calculus Metagenomic DNA.
(A) mapDamge plot of quality-filtered reads mapped to the human genome. These data display characteristic ancient terminal end read damage treated with partial UDG treatment. (B) Principle coordinate analysis of weighted unifrac distances for the 16s rRNA gene. St.LI calculus clusters with ancient tooth roots, and away from soil and gut profiles.

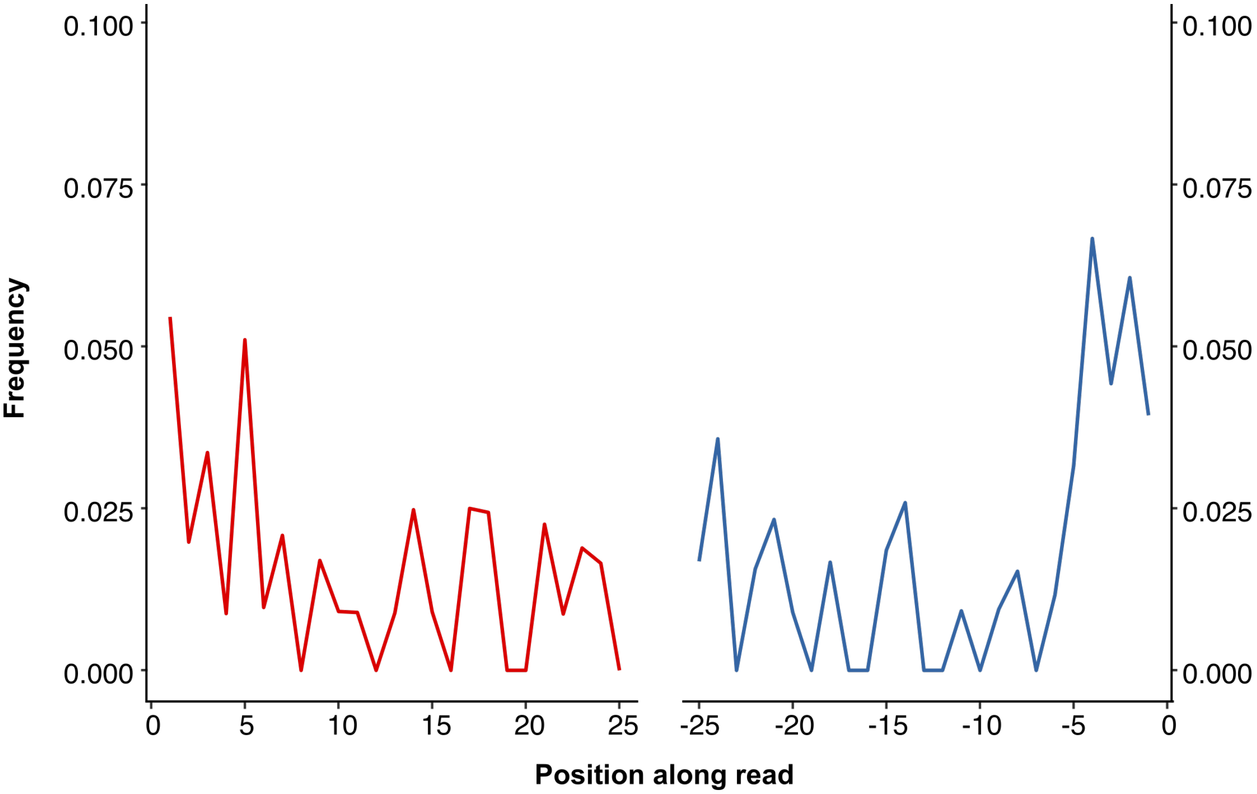

Supplementary Fig. 9 Damage plots of reads mapping to *Mycobacterium tuberculosis*. mapDamage plot of shotgun reads mapped to the ancestral *Mycobacterium tuberculosis* reference genome[6]. The erratic pattern illustrates the uncertainty to authenticate the 402 unique reads mapping to *M. tuberculosis*. *M. tuberculosis* DNA is more commonly recovered in historical and ancient samples near active lesions and tooth pulp[7,8].

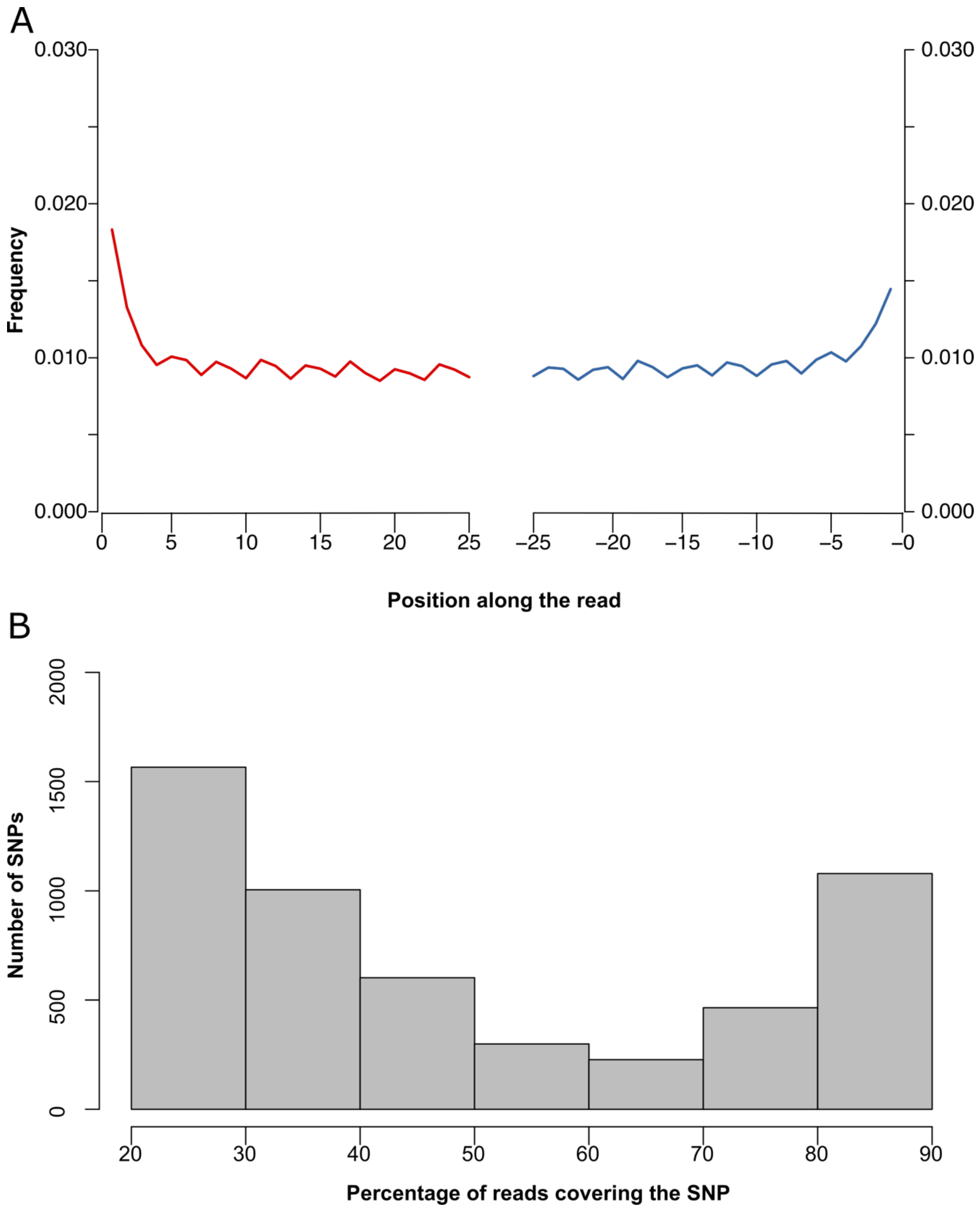

Supplementary Fig. 10 Damage plots and heterozygous SNP frequency plots for *Acinetobacter junii*.

(A) mapDamage plot of reads mapping to *A. junii*. (B) Histogram of heterozygous SNP frequencies for *A. junii*.

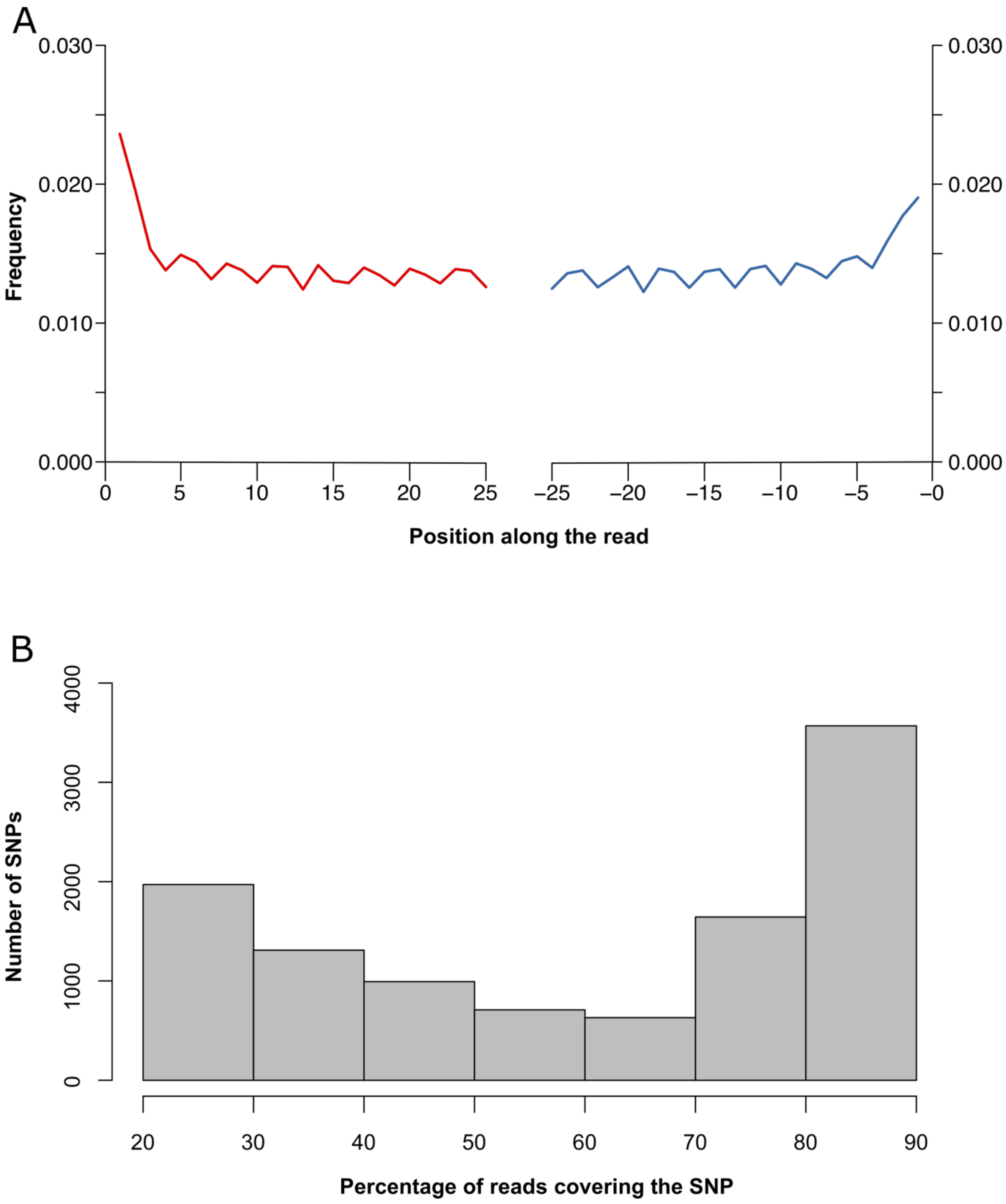

Supplementary Fig. 11 Damage plots and heterozygous SNP frequency plots for *Acinetobacter nosocomialis*.

(A) mapDamage plot of reads mapping to *A. nosocomialis*. (B) Histogram of heterozygous SNP frequencies for *A. nosocomialis*.

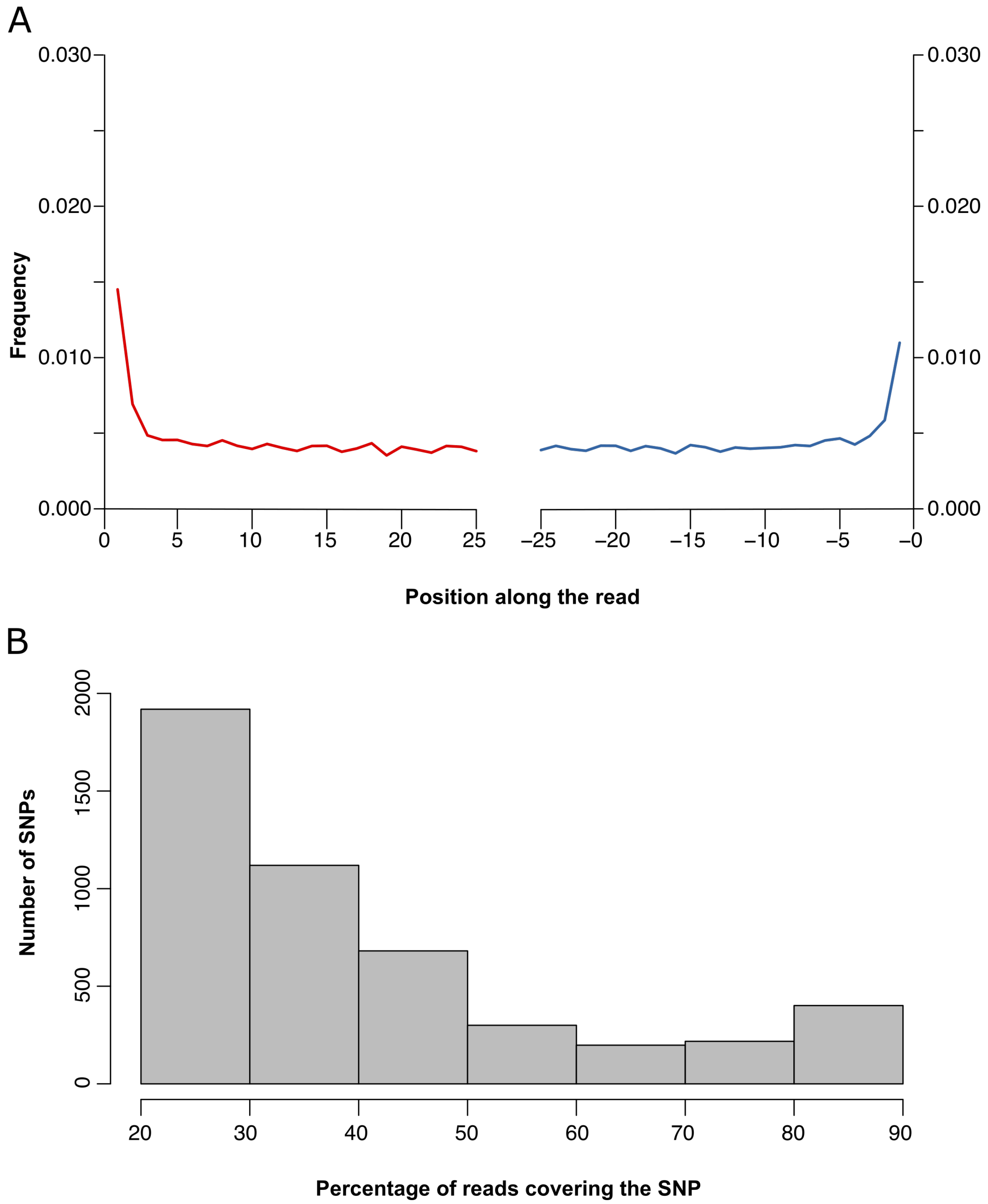

Supplementary Fig. 12 Damage plots and heterozygous SNP frequency plots for *Klebsiella pneumoniae*.

(A) mapDamage plot of reads mapping to *K. pneumoniae*. (B) Histogram of heterozygous SNP frequencies for *K. pneumoniae*.

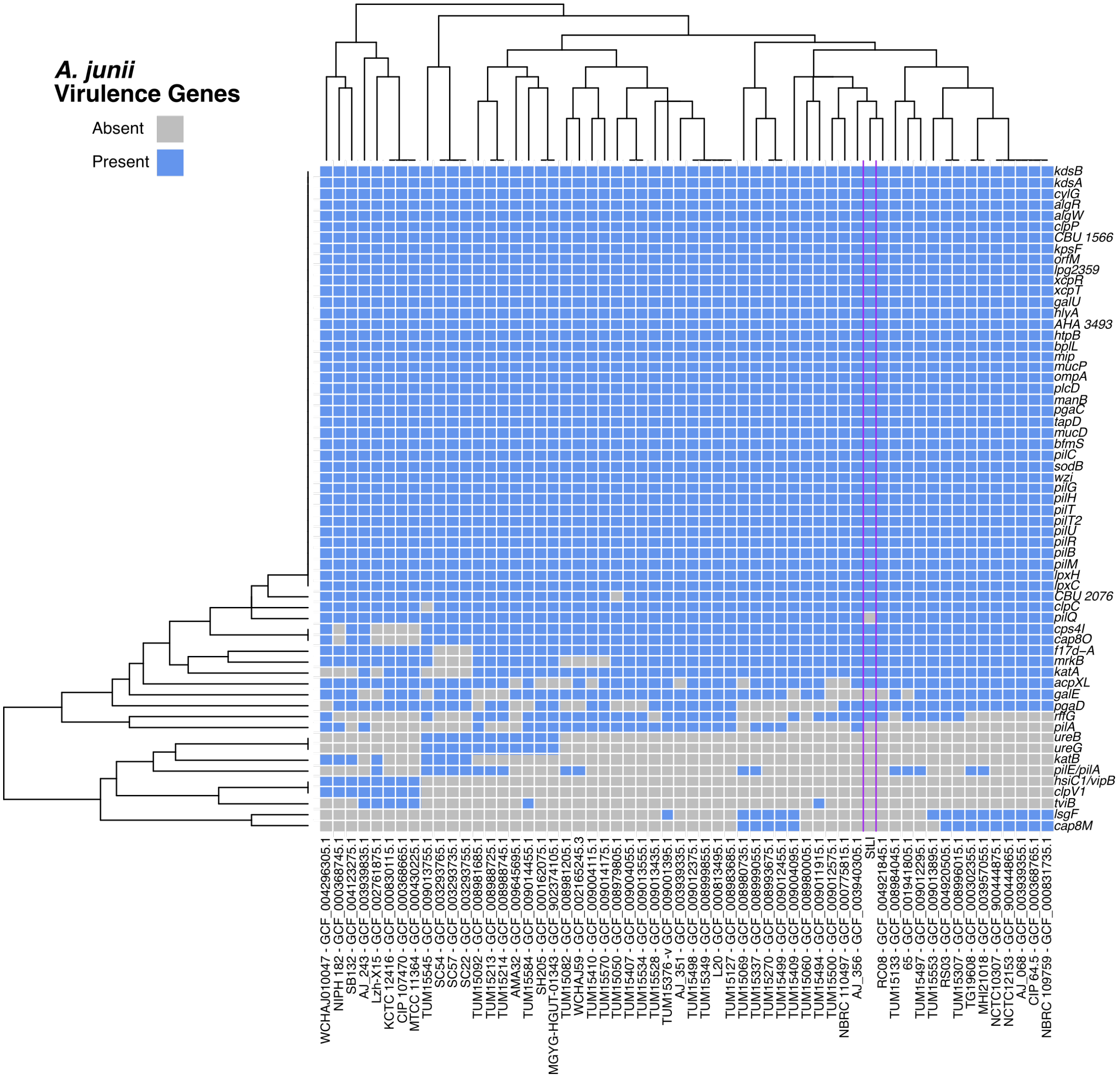

Supplementary Fig. 13 Heatmap of *Acinetobacter junii* virulence genes.
Presence (blue) and absence (gray) *Acinetobacter junii* virulence genes found in St.LI and comparative *A. junii* species genomes.

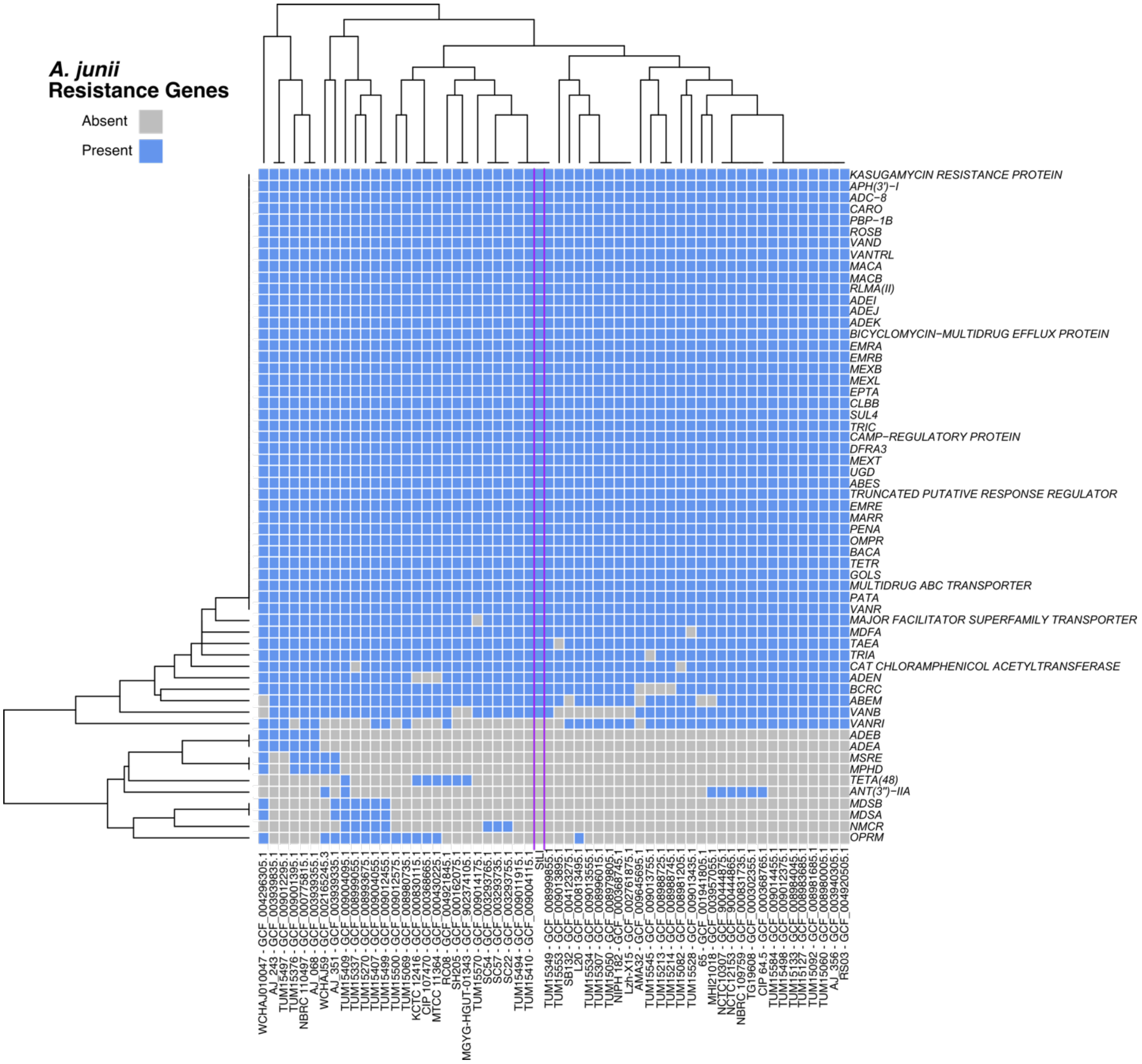

Supplementary Fig. 14 Heatmap of *Acinetobacter junii* resistance genes.
Presence (blue) and absence (gray) *A. junii* resistance genes found in St.LI and comparative *A. junii* species genomes.

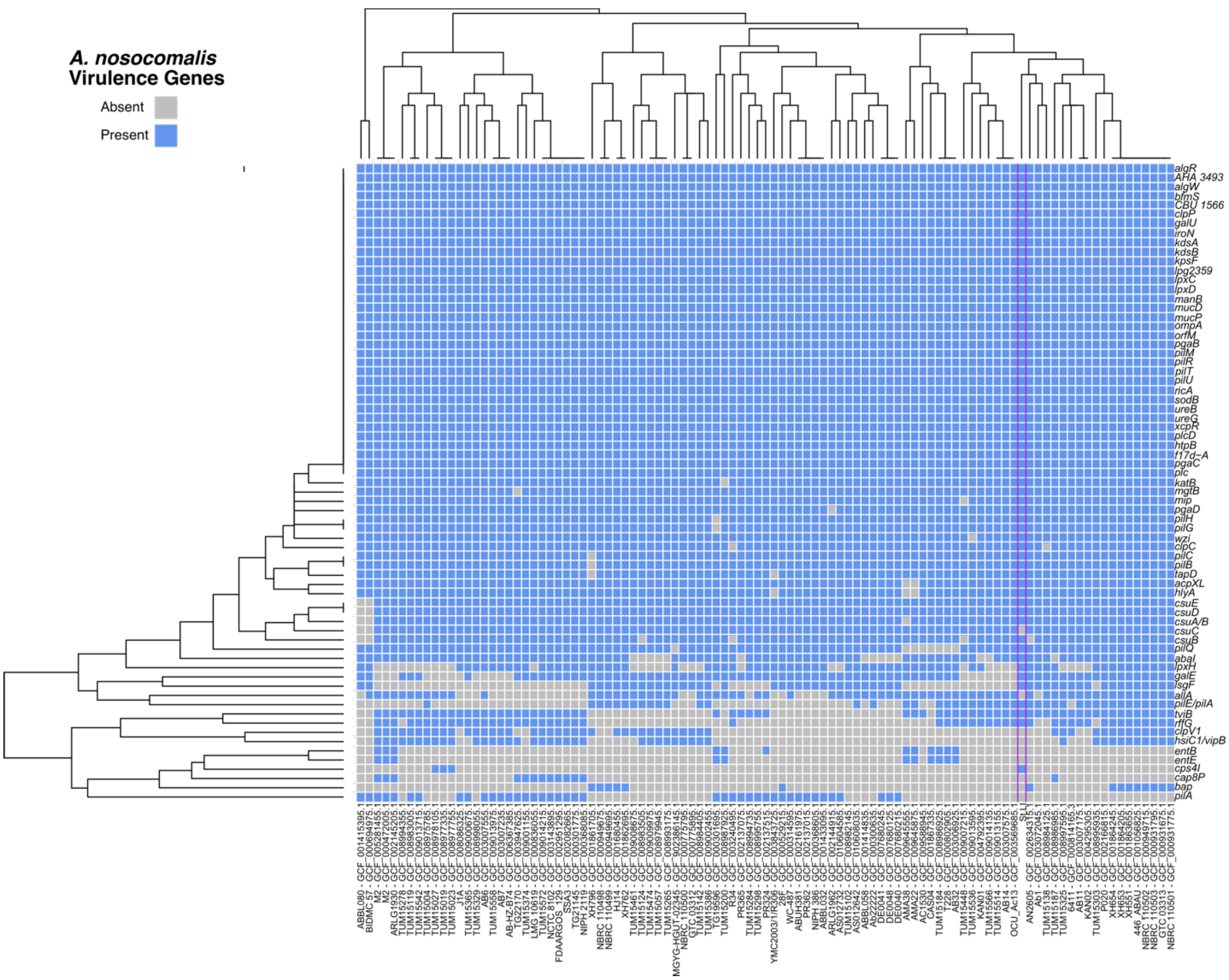

Supplementary Fig. 15 Heatmap of *Acinetobacter nosocomialis* virulence genes.
Presence (blue) and absence (gray) of *Acinetobacter nosocomialis* virulence genes found in St.LI and comparative *A. nosocomialis* species genomes.

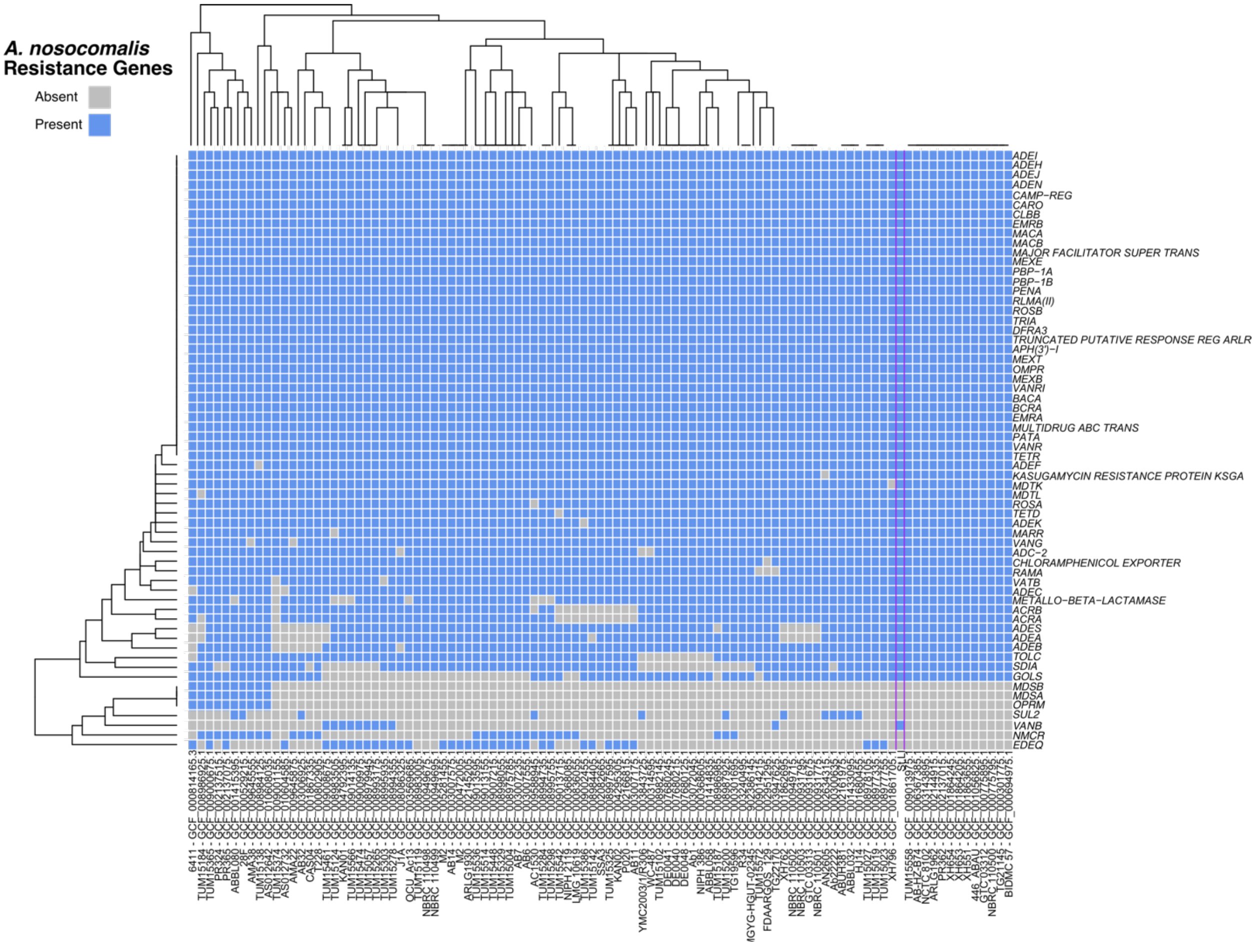

Supplementary Fig. 16 Heatmap of *Acinetobacter nosocomialis* resistance genes.
Presence (blue) and absence (gray) of *Acinetobacter nosocomialis* resistance genes found in St.LI and comparative *A. nosocomialis* species genomes.

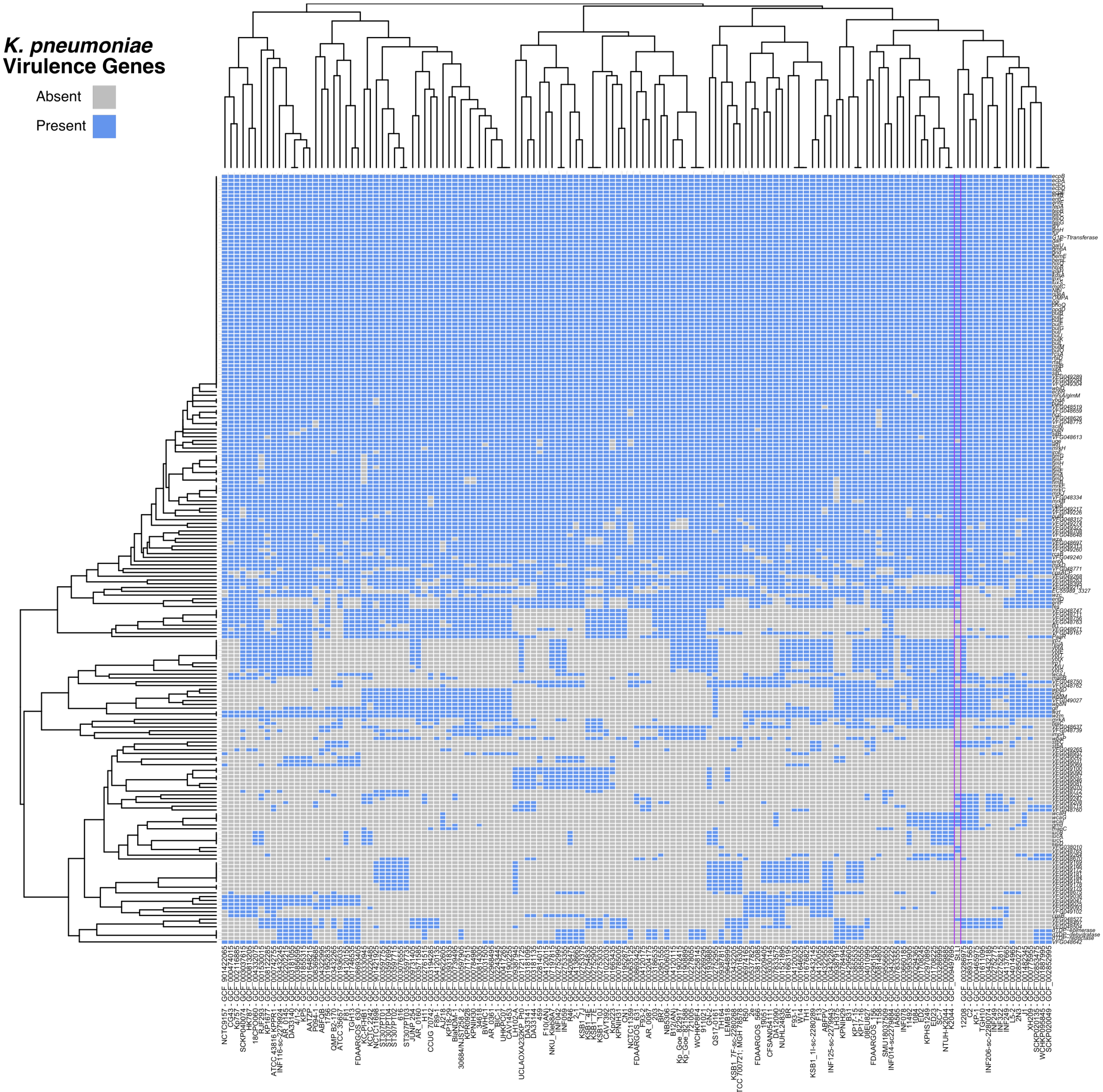

Supplementary Fig. 17 Heatmap of *Klebsiella pneumoniae* virulence genes.
Presence (blue) and absence (gray) of *K. pneumoniae* virulence genes found in St.LI and comparative *K. pneumoniae* species genomes.

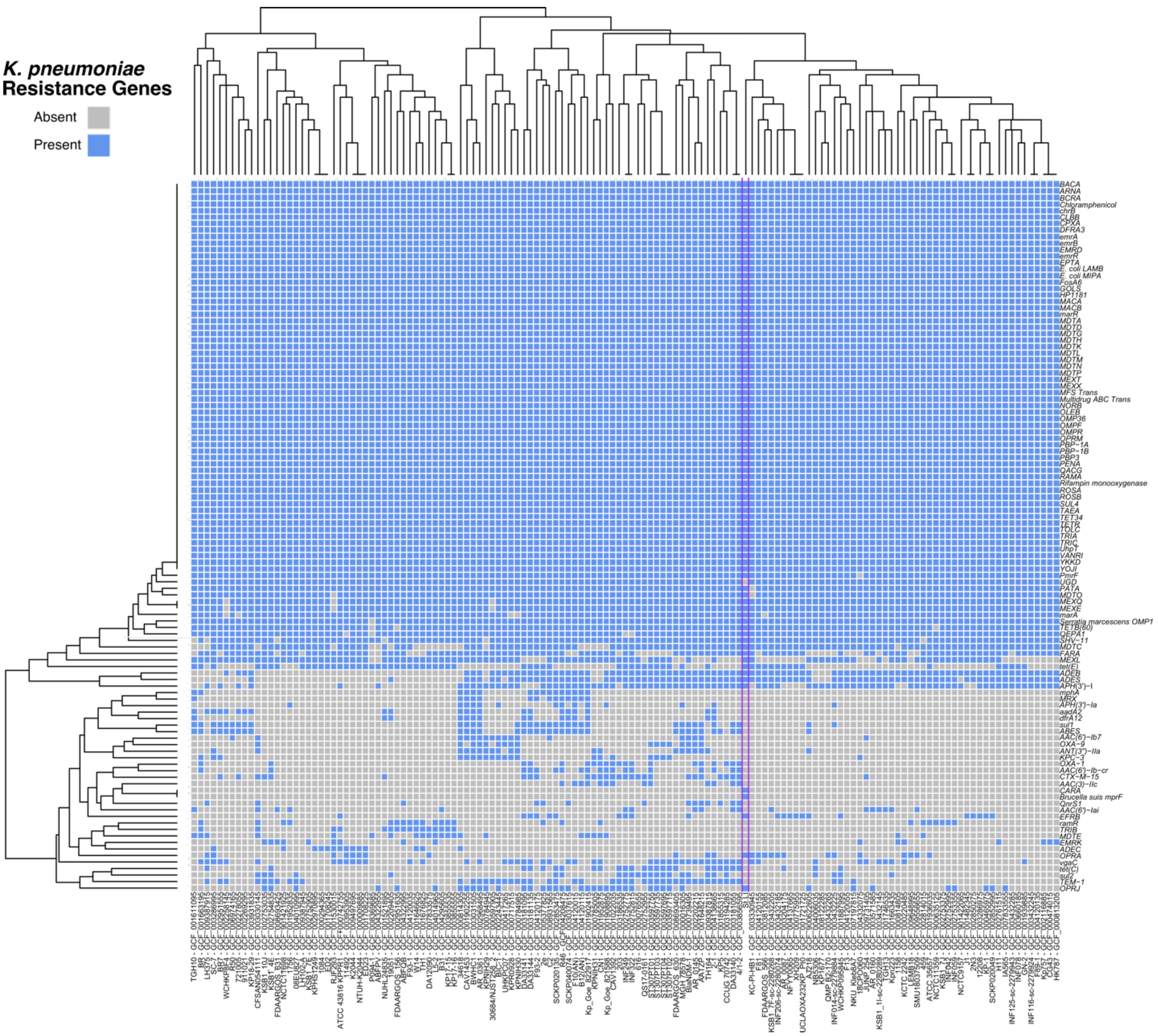

Supplementary Fig. 18 Heatmap of *Klebsiella pneumoniae* resistance genes.

Presence (blue) and absence (gray) of *K. pneumoniae* resistance genes found in St.LI and comparative *K. pneumoniae* species genomes.

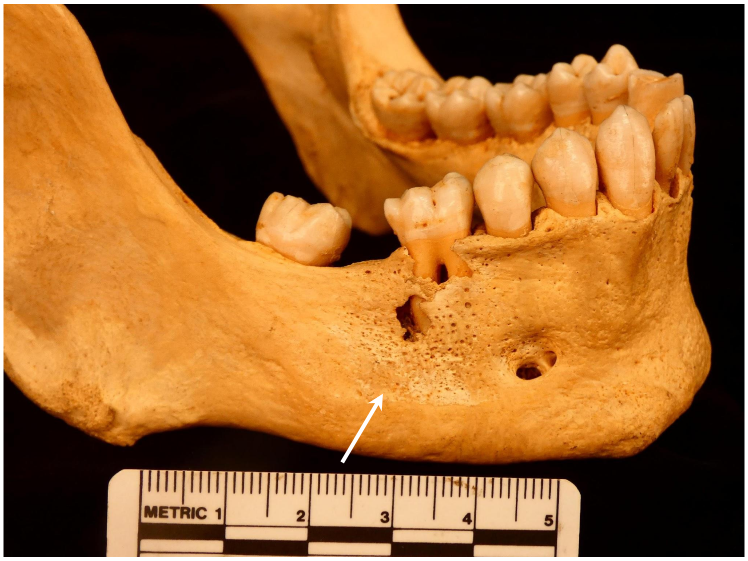

Supplementary Fig. 19 Dental calculus sampling site, right mandibular first molar. The right side of the mandible reveals a draining oral abscess (white arrow) associated with the 1st molar. The sharp margins of this lesion indicate that it was active at time of death.

Supplementary Information References

1. White, D. J. Dental calculus: Recent insights into occurrence, formation, prevention, removal and oral health effects of supragingival and subgingival deposits. *Eur J Oral Sci* **105**, 508–522 (1997).
2. Radini, A., Nikita, E., Buckley, S., Copeland, L. & Hardy, K. Beyond food: The multiple pathways for inclusion of materials into ancient dental calculus. *Am J Phys Anthropol* **162**, 71–83 (2017).
3. White, D. J. Processes contributing to the formation of dental calculus. *Biofouling* **4**, 209–218 (1991).
4. Akcalı, A. & Lang, N. P. Dental calculus: The calcified biofilm and its role in disease development. *Periodontol 2000* **76**, 109–115 (2018).
5. Dangvard Pedersen, D., Milner, G. R., Kolmos, H. J. & Boldsen, J. L. The association between skeletal lesions and tuberculosis diagnosis using a probabilistic approach. *Int J Paleopathol* **27**, 88–100 (2019).
6. Comas, I. *et al.* Human T cell epitopes of Mycobacterium tuberculosis are evolutionarily hyperconserved. *Nat Genet* **42**, 498 (2010).
7. Sabin, S. *et al.* A seventeenth-century Mycobacterium tuberculosis genome supports a Neolithic emergence of the Mycobacterium tuberculosis complex. *bioRxiv* (2019).
8. Harkins, K. M. *et al.* Screening ancient tuberculosis with qPCR: Challenges and opportunities. *PhilosT R Soc B* **370**, 20130622 (2015).
