# Supplementary material for: Remembering St. Louis Individual—structural violence and acute bacterial infections in a historical anatomical collection
Source: Commun Biol. 2022 Oct 3;5:1050. doi: 10.1038/s42003-022-03890-z (PMC9527723; doi:10.1038/s42003-022-03890-z)
Supplement: Supplementary file 2 — Description of Additional Supplementary Files [file 42003_2022_3890_MOESM2_ESM.pdf]

Description of Additional Supplementary Files

File name: Supplementary Data 1
Description: Musculoskeletal activity markers

File name: Supplementary Data 2
Description: Traumatic lesions and 'other' pathological lesions not directly associated with Tuberculosis. Examination followed established descriptions and criteria for pathologies and trauma.

File name: Supplementary Data 3
Description: Observed oral pathologies

File name: Supplementary Data 4
Description: Tuberculosis indicators, following Dangvard Pedersen et al. (2019)*. High diagnostic value indicators are grey.

File name: Supplementary Data 5
Description: Oral microbial community composition of StLI as classified by MetaPhlan3. Sorted by descending percent abundance, the top ten most abundant microbes are known, or can be, opportunistic pathogens.

File name: Supplementary Data 6
Description: Known causative agents of bacterial pneumonia with citations

File name: Supplementary Data 7
Description: Reads mapping to *Mycobacterium tuberculosis*

File name: Supplementary Data 8
Description: PanPhlAn3 gene content analysis

File name: Supplementary Data 9
Description: Mapping statistics for pathogens identified in St.LI

File name: Supplementary Data 10
Description: *Klebsiella pneumoniae* virulence and resistance gene presence/absence analysis

File name: Supplementary Data 11
Description: *Acinetobactoer junii* virulence and resistance gene presence/absence analysis

File name: Supplementary Data 12
Description: *Acinetobactoer nosocomialis* virulence and resistance gene presence/absence analysis

File name: Supplementary Data 13
Description: Reads mapping to the human genome (hg19)

File name: Supplementary Data 14
Description: Reads mapping to GreenGenes (v13.8) database
