# Supplementary material for: Remembering St. Louis Individual—structural violence and acute bacterial infections in a historical anatomical collection
Source: Commun Biol. 2022 Oct 3;5:1050. doi: 10.1038/s42003-022-03890-z (PMC9527723; doi:10.1038/s42003-022-03890-z)
Supplement: Supplementary file 4 — Reporting Summary [file 42003_2022_3890_MOESM4_ESM.pdf]

nature research

Corresponding author(s): Rita M. Austin, COMMSBIO-21-2117

Last updated by author(s): Jul 21, 2022

Reporting Summary

Nature Research wishes to improve the reproducibility of the work that we publish. This form provides structure for consistency and transparency in reporting. For further information on Nature Research policies, see our Editorial Policies and the Editorial Policy Checklist.

Statistics

For all statistical analyses, confirm that the following items are present in the figure legend, table legend, main text, or Methods section.

n/a	Confirmed	
☒	☐	The exact sample size (*n*) for each experimental group/condition, given as a discrete number and unit of measurement
☒	☐	A statement on whether measurements were taken from distinct samples or whether the same sample was measured repeatedly
☒	☐	The statistical test(s) used AND whether they are one- or two-sided<br>*Only common tests should be described solely by name; describe more complex techniques in the Methods section.*
☒	☐	A description of all covariates tested
☒	☐	A description of any assumptions or corrections, such as tests of normality and adjustment for multiple comparisons
☒	☐	A full description of the statistical parameters including central tendency (e.g. means) or other basic estimates (e.g. regression coefficient) AND variation (e.g. standard deviation) or associated estimates of uncertainty (e.g. confidence intervals)
☒	☐	For null hypothesis testing, the test statistic (e.g. *F*, *t*, *r*) with confidence intervals, effect sizes, degrees of freedom and *P* value noted<br>*Give P values as exact values whenever suitable.*
☒	☐	For Bayesian analysis, information on the choice of priors and Markov chain Monte Carlo settings
☒	☐	For hierarchical and complex designs, identification of the appropriate level for tests and full reporting of outcomes
☒	☐	Estimates of effect sizes (e.g. Cohen's *d*, Pearson's *r*), indicating how they were calculated

Our web collection on statistics for biologists contains articles on many of the points above.

Software and code

Policy information about availability of computer code

Data collection	*Provide a description of all commercial, open source and custom code used to collect the data in this study, specifying the version used OR state that no software was used.*
Data analysis	Please see the Materials and Methods section.

For manuscripts utilizing custom algorithms or software that are central to the research but not yet described in published literature, software must be made available to editors and reviewers. We strongly encourage code deposition in a community repository (e.g. GitHub). See the Nature Research guidelines for submitting code & software for further information.

Data

Policy information about availability of data

All manuscripts must include a data availability statement. This statement should provide the following information, where applicable:

- Accession codes, unique identifiers, or web links for publicly available datasets
- A list of figures that have associated raw data
- A description of any restrictions on data availability

The NGS sequencing data that support the findings of this study have been archived in NCBI Sequence Read Archive (SRA) under the BioProject ID PRJNA851947. Human DNA sequences have been removed from the available SRA data to protect the genetic identity and potential genealogical descendants of St.LI. The data used for pathogen analyses are available in Supplementary Tables 10-12.

Field-specific reporting

Please select the one below that is the best fit for your research. If you are not sure, read the appropriate sections before making your selection.

☐ Life sciences ☒ Behavioural & social sciences ☐ Ecological, evolutionary & environmental sciences

For a reference copy of the document with all sections, see nature.com/documents/nr-reporting-summary-flat.pdf

Behavioural & social sciences study design

All studies must disclose on these points even when the disclosure is negative.

Study description	Interdisciplinary assessment of documented cause of death for a single individual in a historical reference collection using qualitative, metagenomic, and historical documentation.
Research sample	A single individual, St. Louis Individual, from the Robert J. Terry Anatomical Collection at the National Museum of Natural History is discussed in this case study. The cause of death (CoD) for Terry Collection individuals is documented in death certificates, allowing for molecular evidence of CoD to be cross-referenced.
Sampling strategy	St. Louis Individual was identified due to the breadth and depth of data and interpretations that could be made.
Data collection	Metagenomic data was generated using Illumina sequencing, skeletal data was gathered on recording forms, and historical information was gained from archives, books, newspapers, etc.
Timing	Data was continuously collected.
Data exclusions	Metagenomic sequences identified as human were limited from in-depth analysis in this study.
Non-participation	None dropped out.
Randomization	No experimental groups were used.

Reporting for specific materials, systems and methods

We require information from authors about some types of materials, experimental systems and methods used in many studies. Here, indicate whether each material, system or method listed is relevant to your study. If you are not sure if a list item applies to your research, read the appropriate section before selecting a response.

Materials & experimental systems

n/a	Involved in the study	
☒	☐	Antibodies
☒	☐	Eukaryotic cell lines
☐	☒	Palaeontology and archaeology
☒	☐	Animals and other organisms
☒	☐	Human research participants
☒	☐	Clinical data
☒	☐	Dual use research of concern

Methods

n/a	Involved in the study	
☒	☐	ChIP-seq
☒	☐	Flow cytometry
☒	☐	MRI-based neuroimaging

Palaeontology and Archaeology

Specimen provenance	Access was permitted by the Smithsonian Institution's National Museum of Natural History.
Specimen deposition	The individual's skeletal remains are currently housed in the National Museum of Natural History
Dating methods	Archival documentation

☐ Tick this box to confirm that the raw and calibrated dates are available in the paper or in Supplementary Information.

Ethics oversight	No ethical guidelines exist for the scientific use of deceased individuals. Instead, we have endeavored to openly discuss the ethical issues surrounding the historic documented collection, as well as why and how individuals were incorporated into the collection.

Note that full information on the approval of the study protocol must also be provided in the manuscript.
